# The Bright and Dark Side of Gossip for Cooperation in Groups

**DOI:** 10.3389/fpsyg.2019.01374

**Published:** 2019-06-20

**Authors:** Terence D. Dores Cruz, Bianca Beersma, Maria T. M. Dijkstra, Myriam N. Bechtoldt

**Affiliations:** ^1^Department of Organization Sciences, Vrije Universiteit Amsterdam, Amsterdam, Netherlands; ^2^Department of Management and Economics, EBS Universität für Wirtschaft und Recht, Oestrich-Winkel, Germany

**Keywords:** gossip, cooperation, group protection, emotion venting, groups, teams, short-term, long-term

## Abstract

Recent experimental studies seem to concur that gossip is good for groups by showing that gossip stems from prosocial motives to protect group members from non-cooperators. Thus, these studies emphasize the “bright” side of gossip. However, scattered studies point to detrimental outcomes of gossip for individuals and groups, arguing that a “dark” side of gossip exists. To understand the implications of gossip for cooperation in groups, both the dark and bright side of gossip must be illuminated. We investigated both sides of gossip in two scenario studies. In Study 1 (*N* = 108), we confronted participants with a free-rider in their group and manipulated whether the gossip recipient was the free-rider’s potential victim or not. Participants showed a higher group protection motivation in response to gossip when imagining gossiping to a potential victim of a norm violator compared to a non-victim. They showed a higher emotion venting motivation when imagining gossiping to a non-victim compared to a potential victim. Both these gossip motives were related to an increased tendency to gossip. In Study 2 (*N* = 104), we manipulated whether participants were the targets or observers of gossip and whether the gossip was true or false. Results showed that targets of negative gossip intended to increase their work effort in the short run, but only when the gossip was true. Furthermore, gossip targets reported lower long-term cooperative intentions toward their workgroup regardless of gossip veracity. This paper demonstrates that gossip has both a “dark” and “bright” side and that situational factors and agent perspectives determine which side prevails.

## Introduction

Gossip, defined here as informally exchanging evaluative information about absent third parties, is often perceived as despicable as well as untrustworthy behavior and is condemned as a norm violation in almost all cultures (Wilson et al., 2000; Foster, 2004). For example, Judaism, Christianity, and Islam alike explicitly describe (negative) gossip, often touted as backbiting, as a very severe sin in multiple passages of their respective holy texts (e.g., Leviticus 19:16; Quran 49:12; Romans 1:28–32).

Despite this largely negative perception, several scholars have argued that people are especially interested in sharing and receiving gossip, and spend a considerable amount of their conversations gossiping (Wilson et al., 2000; Foster, 2004; McAndrew, 2008). Indeed, gossip is observed frequently across different types of groups ranging from small-scale hunter-gatherer societies representative of our evolutionary history (Guala, 2012; Von Rueden, 2014) to teams in modern organizations (Kniffin and Wilson, 2005; Grosser et al., 2012). Thus, gossip seems to be a paradoxical phenomenon: It is condemned, but it is widespread.

Strengthening the paradox even further, accumulating evidence from a recent body of literature across scientific disciplines shows that gossip could play a positive role in groups. Specifically, findings demonstrate gossip could play an important role in clarifying group norms, protecting group members from norm violators, and maintaining social order (Nowak and Sigmund, 2005; Kniffin and Wilson, 2010; Beersma and Van Kleef, 2012; Wu et al., 2016a; Fehr and Sutter, 2019). A plethora of recent experimental laboratory studies demonstrate that gossip is motivated by the desire to protect others from people who violate norms of cooperation. Moreover, these studies also demonstrate that when groups gossip, or when simply the possibility exists that they might gossip, this deters group members from behaving selfishly, evidenced by increased donations to partners, offers in dictator games, and contributions in public goods dilemmas (Sommerfeld et al., 2007; Beersma and Van Kleef, 2011; Feinberg et al., 2012b; 2014; Wu et al., 2016a).

As such, across disciplines, our current scientific understanding seems to converge upon the idea that gossip might be beneficial for groups. Indeed, in organization sciences, Brady et al. (2017) recently argued gossip should no longer be regarded as “deviant behavior.” This stands in stark contrast to people’s perceptions of gossip, and demonstrates there may be a “bright” side of gossip in groups, in that gossip is functional by increasing cooperation.

However, at the same time, some findings indicate that gossip is anything but a noble, prosocial act and that it can have detrimental consequences for group members’ feelings and attitudes. McAndrew et al. (2007) found that gossip can be used to selfishly increase one’s status by damaging the reputation of rivals, for example when sharing information that makes a rival less desirable when competing for mates (Wyckoff et al., 2018). Furthermore, several cross-sectional field studies suggest that gossip in real-world organizational contexts may have detrimental consequences for both teams and individual group members. Specifically, gossip in groups has been found to relate to decreased intra-team trust, psychological safety, and viability (Melwani, 2012), to increased negative self-conscious emotions such as fear (Martinescu et al., 2014), to lower work engagement (Georganta et al., 2014), to lower organizational citizenship behavior (i.e., discretionary prosocial behavior within an organization, Organ, 1988) and proactive behavior as well as higher emotional exhaustion (Kong, 2018; Xie et al., 2018; Wu et al., 2018). These findings seem to correspond with lay people’s negative views of gossip and may illustrate the existence of a “dark” side of gossip in groups (e.g., Paine, 1967; Melwani, 2012).

In summary, studies on gossip currently show evidence of both positive and negative motives for gossip as well as positive and negative outcomes of gossip in groups. This mixed evidence demonstrates that we currently do not have a clear image of why people gossip. Moreover, the divergent conclusions of previous studies about the consequences of gossip for groups make it difficult, if not impossible, to answer questions about when and why gossip is functional or dysfunctional for groups. As such, based on the current state of science, it is not possible to advise practitioners, groups and individuals, who are confronted with gossip, on how to deal with it. Therefore, it is important to build theory that helps us understand when and why the dark or bright side of gossip prevails.

The current paper contributes to building such theory. Specifically, when examining the studies on the bright and dark sides of gossip reviewed before, we noted that they suffer from several flaws. First, the majority of studies that point to the “dark side”– to potentially negative effects of gossip – are cross-sectional in nature. The strength of these studies is that most of them were conducted in organizations and real-world teams, enabling the study of gossip as it occurs in the field. However, they do not allow causal conclusions to be drawn (i.e., does gossip cause negative group processes or do negative group processes cause gossip?).

In contrast, studies that point to the “bright side” – to potentially prosocial motives underlying gossip behavior and to positive effects of gossip – are all laboratory experiments or simulations (e.g., Nowak and Sigmund, 2005). Experimental designs and simulations have the important advantage of allowing causal conclusions to be drawn, including the underlying motives for gossip and the effects of gossip. However, these studies also suffer from several issues hindering our understanding of when and why the “bright” or “dark” side of gossip prevails.

First, previous studies on the “bright,” prosocial side of gossip have always been designed in a way that they can only shed light on gossip that serves a social function. These studies usually confront participants with a free-rider, an individual that does not contribute to group efforts, yet reaps group rewards at the detriment of the other group members (e.g., Beersma and Van Kleef, 2011; Feinberg et al., 2014). Then, they create the option of gossiping about this with someone who could be a potential victim of the free-rider in the future. In case participants do so, it will be considered evidence that gossip results from prosocial motivation – the drive to help others and protect them from norm violators. For example, Feinberg et al. (2012b) showed that people gossiped to protect group members from free-riders even when engaging in gossip came at a personal cost. They concluded that prosocial motivation activates people to gossip in order to protect group members.

Although we do not argue that prosocial motives cannot drive gossip behavior, it is important to note that the described experiments on prosocial gossip always create a situation in which the person to whom the gossip is directed could potentially suffer from the free-rider’s behavior if the gossip did not warn them about this. However, studies in which participants can exclusively gossip to potential victims of norm violators trivialize how motives other than group protection could motivate their gossip. In real life, people might have many more motives to gossip than just group protection, and these motives may include both prosocial and selfish ones. Therefore, the first goal of this paper was to demonstrate that different motives can drive gossip in different situations.

A second issue that limits our current understanding of gossip is that previous experimental studies ignored the perspective of the person being gossiped about. Whereas the threat of gossip might motivate group members to “stay in line” and cooperatively behave in accordance with group norms, being the target of gossip is likely a painful experience for targets. On top of this, targets are often aware that others gossiped about them. A recent study found that 73% of the respondents were able to recall gossip they had heard about themselves; predominantly they had heard it from the recipients, or they had learned it accidentally (Martinescu, 2017). Previous experimental research often suggested that participants *could become* the target of gossip (e.g., Beersma and Van Kleef, 2011), but did not directly examine how people intend to behave when they *do* become gossip targets (see also Martinescu et al., under review). For example, group cooperation might deteriorate when group members – instead of feeling threatened to become a target of gossip – actually become targets of gossip because it might signal a lack of inclusion in the group. Perceiving a lack of inclusion has been found to predict more aggressive and less prosocial behavior (Twenge et al., 2007; DeWall et al., 2012; Jansen et al., 2014), and this is in stark contrast to the positive effects of gossip on cooperation in groups (Beersma and Van Kleef, 2011; Feinberg et al., 2012b; Wu et al., 2016a). Therefore, including the target’s perspective in gossip research is important to gain a more complete understanding of the effects of gossip on groups. This was the second goal of this paper.

A third issue is that experimental studies have focused on gossip that follows norm violations and that correctly identifies norm violators. In these cases, the information exchanged about the absent third party corresponds to reality in the sense that it truly and genuinely describes targets’ non-cooperative behavior. Yet, in real life, gossip might also be false. Gossipers may for example state that targets behaved uncooperatively whereas actually, they did not. To fully understand the consequences of gossip in groups, we would have to incorporate false gossip in experimental studies, and this was the third goal of this paper.

A final issue characterizing earlier experimental studies on the “bright” side of gossip is that they have exclusively examined behavior in short-term cooperation tasks as dependent variables. Specifically, these studies examined whether gossip increases cooperative decisions in short-term public goods dilemmas or dictator games as a direct response to the threat of gossip (see for example Beersma and Van Kleef, 2011; Wu et al., 2016a). In real-life contexts such as the workplace, group processes entail more than short-term cooperation decisions; longer-term willingness to cooperate also presents an important outcome. Such long-term cooperation can become visible in organizational groups in the form of organizational citizenship behavior (a person’s voluntary prosocial behavior within an organization that is not part of his or her contractual tasks; e.g., Organ, 1988; Kong, 2018), or commitment to group goals in the long run. Whereas gossip might increase group members’ tendency to comply with group norms in the short run, in the long run it might alienate targets from the group and thus reduce their cooperation. To really understand the consequences of gossip in groups, we would, therefore, have to incorporate broader measures of cooperation in experimental studies, and this was the fourth goal of this paper.

In order to meet the goals we described above, we ran two scenario studies with student participants. For our first goal (increasing understanding of the motives that drive gossip), we designed a study to test whether individuals would gossip to protect their group members, but also whether there are other motives underlying their tendency to gossip. The second study was designed to test the influence of true and false gossip on several indicators of cooperation in groups; here, we analyzed both gossip targets and observers (the second, third, and fourth goals of this paper). Taken together, these studies further our understanding of gossip in groups, specifically what drives this behavior and how gossip can serve or harm the interests of groups. Furthermore, these studies provide broad insight not only into the gossip sender’s perspective but also into the less frequently studied target’s perspective and thus paint a more complete picture of gossip in groups.

## Study 1

Previous studies on “prosocial gossip” always studied gossip in situations in which gossip senders could exclusively gossip to recipients who were potential victims of the behavior of the target of gossip (see e.g., Feinberg et al., 2012b). Therefore, in studies on the “bright” side, the group protection motive always applies prominently to gossip whereas other motives are constrained to be of lesser importance.

In real life, people can gossip for reasons other than protecting potential victims of norm violators. Beersma and Van Kleef (2012) distinguish additional motives (see also Foster, 2004; Beersma et al., 2019). First, they argue that gossip can serve a social enjoyment motive: wanting to have fun with others and distracting or stimulating oneself during daily activities can motivate gossiping (Foster, 2004; Beersma and Van Kleef, 2012). Gossip can also be driven by the motivation to gather and validate information; via gossip, people can acquire new information about a gossip target, validate their opinion of the gossip target, engage in social comparison, and thus, map their social world (Rosnow, 1977; Wert and Salovey, 2004; Beersma and Van Kleef, 2012). Moreover, gossip may be motivated by a “negative influence motive” akin to indirect aggression; gossip senders may engage in gossip to negatively manipulate others’ opinions of a gossip target to benefit themselves (Archer and Coyne, 2005; McAndrew et al., 2007; Beersma and Van Kleef, 2012). Finally, several studies indicate gossip can be motivated by a desire to ventilate emotions: Gossiping might help gossip senders to get rid of strong feelings related to the gossip target (Waddington and Fletcher, 2005; Feinberg et al., 2012b; Grosser et al., 2012).

If gossip indeed serves as a mechanism to protect groups against norm violators, as experimental studies on prosocial gossip argue (Feinberg et al., 2012b; Wu et al., 2016a), we would expect to observe a higher tendency to gossip in situations in which gossip might help to protect a group member from another group member’s norm violation than in situations in which this is not possible. The latter situation, however, has to be taken into account to examine whether the other potential motives to engage in gossip discussed above also play a role.

In line with previous studies on prosocial gossip, we expect a situation in which there is the opportunity to gossip to a potential victim of a free-rider to lead to a higher tendency to gossip than a situation in which one can gossip to someone who is not a potential victim (H1a). Furthermore, we expect the group protection motive to be activated more strongly in the former situation than in the latter (H1b). Finally, we expect the group protection motive to mediate the effects of the gossip recipient (potential victims of the free-rider or not) on the tendency to gossip (H1c). We do not make predictions for the other motives to gossip. However, we explore the effect of these motives as well as the effect of having the opportunity to gossip to a potential victim or not through these motives, thus contributing to a more complete understanding of what motivates gossip in different situations.

### Methods

#### Participants

A total of 108 students (69% female) at a large public university (75.9%) or university of applied sciences in the Netherlands were recruited personally, via email, and via social media to participate in a single factor between-subjects design on gossiping. Participants read a scenario in which we described that they were confronted with a free-rider (see *Procedure and Scenarios* below). Fifty-five participants (69.1% female, 74.5% public university, *M*_age_ = 23.27, *SD*_age_ = 4.11, Range_age_ = 18–38) were assigned to the “victim condition.” They were led to believe that the gossip recipient was a potential victim of the free-rider (see Procedure and Scenarios for a full description of the manipulation). Fifty-three participants (67.9% female, 77.4% public university, *M*_age_ = 23.77, *SD*_age_ = 5.71, Range_age_ = 19-55) were assigned to the “non-victim condition,” where they were led to believe that the recipient was not a potential victim of the free-rider. There were no sociodemographic differences between the conditions (*p*s > 0.601, Cohen’s *d*s < 0.08; Cohen, 1988).

#### Procedure and Scenarios

Participants were asked in person, or via email, Facebook or LinkedIn, if they wanted to participate in a study of people’s reactions in social situations (this was done to reduce negative associations with the term gossip). If they indicated that they wished to participate, they received a link to an online questionnaire, presented in Dutch. Then, participants completed a questionnaire assessing their social value orientation (SVO) (see *Measures* below). Second, participants read a scenario in which they had to imagine working on a study project in a group of four students. The group project consisted of two assignments (task 1, task 2) and their presentations (task 3, task 4). This group project required a passing grade in order to graduate and take a previously planned holiday. Their group had decided to form two pairs that would each carry out two tasks. Each pair would first work together on one of the four tasks and then switch partners for a final (second) task. In that way, every student had to perform two out of four tasks. In the first task, the participant worked with a fellow student who was constantly slacking off, causing them to have to compensate for the fellow student’s carelessness and sloppy work. In the victim condition, participants then had to imagine meeting a student from the other pair of their group that would soon be paired with the slacking student for the second and final task. In the non-victim condition, participants were instead informed that they had to imagine meeting another student who was not a member of their student group and who would not be coupled with the slacking student or the participant for any assignment. Participants were randomly assigned to one of two conditions. After reading the scenario, participants indicated their tendency to gossip, followed by their motives to gossip. Subsequently, participants provided their demographics, thoughts about the goal of the study, and whether anything was unclear. Excluding participants who indicated confusion about the scenario did not change the results reported below, therefore they were included. Most participants mentioned they had no idea or did not reply and the rest of the participants indicated that the study involved conflict and cooperation as fitting with the recruitment instructions. Finally, participants were debriefed about the true purpose of the study with regard to gossip behavior and thanked again for their participation in the study. The study protocol was approved by the ‘Research Ethics Review Committee (RERC)’ of the Social Sciences faculty at the Vrije Universiteit Amsterdam.

#### Measures

##### Tendency to gossip

Participants rated their tendency to gossip about their work partner who was slacking off in this scenario, on seven items [e.g., “To what extent is it likely that you would talk about group member (A) with this person?”; adapted from Beersma and Van Kleef, 2012] using a 7-point Likert scale (1 = *very unlikel*y to 7 = *very likely*). Internal consistency was α = 0.74.

##### Motives to gossip

Participants were asked to rate their motives to engage in gossiping using an extended version of the Motives to Gossip questionnaire (Beersma and Van Kleef, 2012; Dores Cruz et al., 2019). The extended questionnaire consists of 31 items measuring five dimensions using a seven-point Likert scale ranging from 1 (*completely disagree*) to 7 (*completely agree*). The *social enjoyment* motive was measured with five items (e.g., “For me, a reason to instigate this conversation was to spend time with the recipient in an enjoyable manner.”; α = 0.83). The *negative influence* motive was measured with five items (e.g., “For me, a reason to instigate this conversation was to put the third person in a negative light.”; α = 0.83). The *information gathering and validation* motive was measured with nine items (e.g., “For me, a reason to instigate this communication was to check whether the recipient had the same ideas about the target.” α = 0.95). The *group protection* motive was measured with five items (instead of three used in the original version by Beersma and Van Kleef (2011); e.g., “For me, a reason to instigate this conversation was to prevent the recipient from becoming a victim of the target’s behavior.”; α = 0.94). A subscale measuring the motive of *venting emotions* was added (seven items, e.g., “For me, a reason to instigate this conversation was to blow off steam.”; α = 0.91).^1^ Exploratory Principal Components analysis using direct oblimin rotation demonstrated that the items loaded on their intended subscales and all subscales showed good internal consistency.

##### Other measures

Participants were asked to indicate their gender, age, educational level,^2^ which study program they followed, what they thought was the goal of the study, and whether something was unclear. Participants also responded to the Triple Dominance Measure (Van Lange et al., 1997) to measure their SVO. Due to the small sample size and related caution about (un)detected effects (Cohen, 1992; Maxwell, 2004), exploratory analyses including SVO are presented in the Supplementary Material.

Descriptive statistics and intercorrelations for all scales analyzed below are shown in Tables 1, 2, respectively.

**TABLE 1:** Descriptive statistics for tendency and motives to gossip per condition and *post hoc* comparison using Bonferroni corrections.

	**Total**		**Victim**		**Non-victim**		**Mean difference**		
	***M***	***SD***	***M***	***SD***	***M***	***SD***	***t***	***p***	**Cohen’s *d***
Tendency to gossip	4.95	0.92	5.22	0.82	4.62	1.11	3.25	0.002	0.61
**Motives to gossip**									
Information gathering and validation	4.43^bc^	1.24	4.89^bc^	1.35	4.00^bcde^	1.30	3.48	< 0.001	0.67
Social enjoyment	2.87^acde^	1.40	2.75^ade^	1.31	2.99^acd^	1.16	1.03	0.303	–0.19
Negative influence	2.42^abde^	1.11	2.46^ade^	1.14	2.38^abde^	1.09	0.38	0.707	0.07
Emotion venting	4.66^bce^	1.27	4.42^bc^	1.32	4.92^abce^	1.17	2.10	0.038	–0.39
Group protection	4.12^bcd^	1.70	5.07^bc^	1.42	3.14^acd^	1.39	7.14	< 0.001	1.37

*Superscripts indicate within condition comparisons (all *p*s < 0.029). ^a^Differs significantly from information gathering and validation, ^b^differs significantly from social enjoyment, ^c^differs significantly from negative influence, ^d^differs significantly from emotion venting, ^e^differs significantly from group protection.*

**TABLE 2:** Intercorrelations between demographics, dependent, and independent variables (Study 1).

	**1**	**2**	**3**	**4**	**5**	**6**	**7**	**8**	**9**
(1) Tendency to gossip									
**Motives:**									
(2) Social enjoyment	0.12								
(3) Information gathering and validation	$0.23{}^{*}$	$0.21{}^{*}$							
(4) Negative influence	0.08	$0.22{}^{*}$	$0.35{}^{*,**}$						
(5) Emotion venting	$0.31{}^{**}$	$0.20{}^{*}$	0.05	$0.19{}^{*}$					
(6) Group protection	$0.43{}^{*,**}$	< 0.01	$0.36{}^{*,**}$	$0.23{}^{*}$	–0.14				
(7) Victim condition	$0.30{}^{**}$	–0.10	$0.33{}^{*,**}$	0.04	$-0.20{}^{*}$	$0.57{}^{*,**}$			
(8) Gender	–0.03	–0.18	$0.23{}^{*}$	0.03	0.12	0.01	0.01		
(9) Age	–0.13	–0.02	–0.05	$0.30{}^{**}$	–0.05	–0.12	–0.05	–0.18	
(10) Education	$0.32{}^{**}$	–0.01	0.11	0.03	0.12	0.13	–0.03	–0.01	–0.16

Victim condition is coded 1 = victim, 0 = non-victim, SVO is coded 1 = prosocial, 0 = proself, gender is coded 1 = male, 0 = female, education is coded 1 = university of applied sciences, 0 = university. ^*^p < 0.05, ^∗∗^*p* < 0.01, ^∗∗∗^*p* < 0.001.

#### Statistical Analyses

To test Hypotheses 1a, b, and c–that gossiping to potential victims of gossip triggers the group protection motive to gossip, therefore resulting in a higher tendency to gossip in this condition than in the non-victim condition–we computed a series of mediator regression analyses. Specifically, we tested whether the different motives to gossip (information gathering and validation, social enjoyment, negative influence, emotion venting, and group protection) mediated the relationship between (non)victim condition and the tendency to gossip with the software package PROCESS (Hayes, 2017; Model 4).^3^ We tested the significance of the indirect effect with bias-corrected bootstrapping procedures using 10.000 samples. All variables, except for the dummy-coded victim condition, were standardized prior to being entered into the model.

To further explore the difference in motives between the two gossip recipient conditions (victim vs. non-victim), a repeated measures ANOVA with the five motives as a within-subjects factor and victim condition as a between-subjects factor was conducted.

To explore the effects of the motives on the tendency to gossip separately per condition, two linear regression analyses with all motives to gossip as predictors of the tendency to gossip were performed. All variables were standardized prior to entering the analyses.

### Results

The correlations between demographics, victim condition, motives to gossip, and tendency to gossip can be found in Table 2. There was a significant positive association between age and the negative influence motive; older participants indicated gossiping based on this motive to a higher extent than younger participants. Further, there was a significant positive association between gender and the information validation motive indicating that women scored higher on this motive than men [*M_men_* = 3.97, *SD* = 1.19; *M_women_* = 4.65, *SD* = 1.45, *t*(106) = −2.40, *p* = 0.012]. Finally, there was a positive association between education and tendency to gossip; university students showed a higher tendency to gossip (*M* = 5.10, *SD* = 0.90) than university of applied sciences students (*M* = 4.36, *SD* = 1.15), *t*(106) = 3.42, *p* = 0.001.

Further, several gossip motives were positively and significantly associated with one another. This indicates gossip in the scenario is motivated by multiple motives, with some motives being activated together more strongly than others. The social enjoyment motive was positively associated with the information gathering and validation motive, the negative influence motive, and the emotion venting motive. The information gathering and validation motive was positively associated with the negative influence motive and the group protection motive. The negative influence motive was significantly associated with the emotion venting motive and the group protection motive. In contrast to findings by Feinberg et al. (2012b), the group protection motive and the emotion venting motive were unrelated.

#### Hypotheses Tests

**FIGURE 1:**
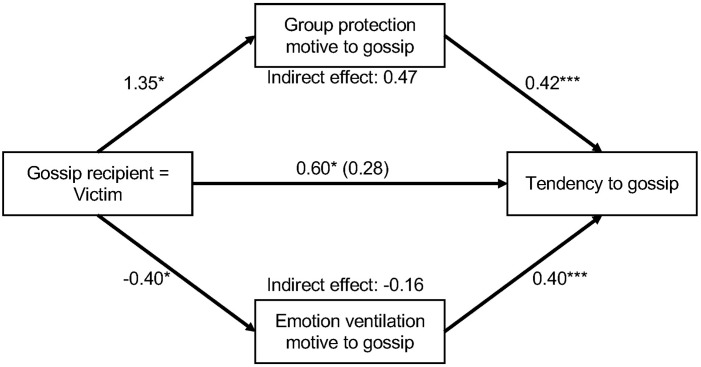
Unstandardized regression coefficients for the relationship between victim condition (victim versus non-victim) and tendency to gossip as mediated by group protection and emotion venting motives. The other motives have been omitted for clarity. The regression coefficient between victim condition and tendency to gossip, controlling for all other motives is in parentheses. Victim condition is dummy-coded (1 = victim, 0 = non-victim). ^*^p < 0.05, ^∗∗∗^*p* < 0.001.

Supporting Hypothesis 1a, victim condition had a large and positive effect on tendency to gossip [Total effect: *b* = 0.60, *SE* = 0.18, *t*(106) = −2.32, *p* = 0.002, Cohen’s *d* = 0.98], indicating that when the recipient of the gossip is a potential victim of the person one is gossiping about, people are more likely to gossip than when the recipient is an observer (see Table 1 and Figure 1).

Supporting Hypothesis 1b, participants in the condition who were led to believe the gossip recipient was a potential victim of the norm-violator (victim condition) as compared to participants who were led to believe the gossip recipient was not a potential victim of the norm violator (non-victim condition) also scored higher on the group protection motive [*b* = 1.35, *SE* = 0.16, *t(*106) = 7.14, *p* < 0.001, Cohen’s *d* = 1.37].

Supporting Hypothesis 1c, when controlling for victim condition and the other motives, the group protection motive [*b* = 0.42, *SE* = 0.10, *t*(101) = 3.06, *p* < 0.001, partial *r^2^* = 0.14] had a large positive relationship with tendency to gossip. After controlling for all motives, the relationship between victim condition and tendency to gossip was weaker and no longer significant [direct effect: *b* = 0.28, *SE* = 0.20, *t*(101) = 1.37, *p* = 0.172, partial *r^2^* = 0.02], indicating that the tendency to gossip to a potential victim of the target is fully mediated by gossip motives. Furthermore, the bootstrapped indirect effect of the group protection motive was statistically significant (*b* = 0.47, *SE* = 0.16, 95% CI: [0.15; 0.83]).

Looking at the other motives to gossip shows that participants in the victim condition as compared to the non-victim condition scored higher on the information gathering and validation motive [*b* = 0.66, *SE* = 0.13, *t(*106) = 3.61, *p* < 0.001, Cohen’s *d* = 0.67]. Participants in the victim condition as compared to the non-victim condition scored lower on the emotion venting motive [*b* = −0.40, *SE* = 0.19, *t(*106) = −2.10, *p* = 0.038, Cohen’s *d* = −0.39]. Victim condition had no significant effect on the social enjoyment motive [*b* = −0.20, *SE* = 0.19, *t(*106) = −1.03, *p* = 0.303, Cohen’s *d* = −0.19] or the negative influence motive [*b* = 0.07, *SE* = 0.19, *t*(106) = 0.38, *p* = 0.310, Cohen’s *d* = −0.07].

When controlling for victim condition and the other motives, the emotion venting motive [*b* = 0.40, *SE* = 0.09, *t*(101) = 4.70, *p* < 0.001, partial *r^2^* = 0.18] had a large positive effect on the tendency to gossip. When controlling for victim condition and the other motives, the social enjoyment motive [*b* = 0.07, *SE* = 0.09, *t*(101) = 0.83, *p* = 0.407, partial *r^2^* = 0.01], information gathering and validation motive (*b* = 0.04, *SE* = 0.09, *t*(101) = 0.45, *p* = 0.651, partial *r^2^* < 0.01], and the negative influence motive [*b* = −0.13, *SE* = 0.09, *t*(101) = −1.39, *p* = 0.168, partial *r^2^* = 0.02] had no significant effect on the tendency to gossip. Only the bootstrapped indirect effect of the emotion venting motive (*b* = −0.16, 95% CI: [−0.32; −0.01]) was statistically significant. Pairwise comparisons of indirect effects showed that the indirect effect of the emotion venting motive was significantly different from the indirect effect of the group protection motive (*b* = −0.63, 95% CI: [−1.04; −0.28]). The indirect effects of the social enjoyment motive (*b* = −0.01, 95% CI: [−0.08; 0.02]), the information gathering and validation motive (*b* = 0.03, 95% CI: [−0.10; 0.16]), and the negative influence motive (*b* = −0.01, 95% CI: [−0.08; 0.05]) were not statistically significant. Overall, these results suggest that it makes a difference to whom the gossip is directed: If the recipient is a potential victim of the person one would gossip about, the likelihood of gossip increases significantly. This is because this context activates the motive to protect a group member from a norm violator. In contrast, if the recipient is not a potential victim of the person one would gossip about, it activates their motive to vent emotion, which also increases people’s tendency to gossip. However, the effect of the latter in explaining the tendency to gossip is weaker.

#### Exploratory Analyses

Investigating the difference in motives between the two gossip recipient conditions (victim vs. non-victim) showed a large significant interaction effect between these factors, *F*(4, 424) = 20.13, *p* < 0.001, ${\text{η}}_{\text{p}}^{\text{2}}$ = 0.16, indicating that the difference among motives depends on whether the recipient of the gossip was a potential victim or not. There was also a large significant effect of the within-subject factor, *F*(4, 424) = 80.92, *p* < 0.001, ${\text{η}}_{\text{p}}^{\text{2}}$ = 0.43, and a medium effect of the between-subjects factor, *F*(1, 106) = 9.24, *p* = 0.003, ${\text{η}}_{\text{p}}^{\text{2}}$ = 0.08. This shows that the motives vary in importance and intensity when the recipient is or is not a potential victim of the target. *Post hoc* tests using a Bonferroni correction are displayed in Table 1. The motive of group protection becomes activated to a stronger extent when gossiping to a potential victim whereas the motive of emotion venting becomes activated more strongly when gossiping to a non-victim. Furthermore, when gossiping to a potential victim of a norm violator the group protection and emotion venting motives are equally activated whereas when gossiping to a non-victim, the desire to vent emotions is activated more strongly than the desire to protect the group.

The effects of the motives on the tendency to gossip separately per condition can be found in Table 3. In both conditions, the motive to protect the group and to vent emotions arose as the only significant predictors of the tendency to gossip with both having a positive effect. However, the magnitude of effect sizes were reversed across conditions: In the victim condition, there was a larger effect of the motive to protect the group (*r*^2^_partial_ = 0.20; large effect) than the motive to vent emotions (*r*^2^_partial_ = 0.09; medium to large effect), whereas the non-victim condition showed a larger effect on the motive to vent emotions (*r*^2^_partial_ = 0.32; large effect) than the motive to protect the group (*r*^2^_partial_ = 0.14; large effect). This indicates that in both situations, the motive to protect the group and the motive to vent emotions are activated and they are related to an increased tendency to gossip. However, if the gossip receiver is a potential victim of the person one would gossip about, group protection more strongly motivates the tendency to gossip than venting emotions, whereas when the receiver is not a potential victim of the person one would gossip about, the emotion venting motive more strongly motivates the tendency to gossip than group protection. In both contexts, it seems social enjoyment is irrelevant as a motive to gossip, as are information gathering and validation or the desire to damage the gossip target’s reputation.

**TABLE 3:** Regression analysis per condition of tendency to gossip on motives to gossip.

**Condition**	***b*(*SE*)**	***t***	***p***	**Partial *r*^2^**	***R*^2^**	***F*(df1, df2)**	***p***
**Victim**							
Total model					0.26	3.46 (5,49)	0.009
Social enjoyment	-0.04⁢(0.10)	-0.38⁢(49)	0.705	< 0.01			
Information gathering and validation	0.01⁢(0.12)	0.09⁢(49)	0.926	< 0.01			
Negative influence	-0.08⁢(0.12)	-0.65⁢(49)	0.517	0.01			
Emotion venting	0.23⁢(0.11)	2.16⁢(49)	0.036	0.09			
Group protection	0.44⁢(0.13)	3.48⁢(49)	0.001	0.20			
**Non-Victim**							
Total model					0.41	6.57 (5, 47)	< 0.001
Social enjoyment	0.24⁢(0.14)	1.72⁢(47)	0.093	0.06			
Information gathering and validation	0.04⁢(0.14)	0.31⁢(47)	0.759	< 0.01			
Negative influence	-0.08⁢(0.13)	-0.57⁢(47)	0.574	< 0.01			
Emotion venting	0.64⁢(0.14)	4.76⁢(47)	< 0.001	0.32			
Group protection	0.39⁢(0.16)	2.42⁢(47)	0.009	0.14			

Table 3 also shows that the variance in tendency to gossip explained by the gossip motives is much larger in the non-victim condition (*R*^2^ = 0.41) than in the victim condition (*R*^2^ = 0.26). Apparently, once activated, a more self-centered motive like emotion venting has a larger impact than prosocial motivation to gossip. These results suggest that gossiping can be a coping strategy to deal with negative experiences that is used even when it does not directly benefit someone else.

### Discussion

Our first study aimed to investigate whether people gossip to protect their group members from norm violators and whether other motives also play a role in the tendency to gossip. Supporting our hypothesis, participants displayed a higher tendency to gossip to recipients who were a potential victim of a norm violator than to recipients who were not a potential victim of this person. This effect was mediated by the motivation to protect one’s group against norm violators.

Exploratory analyses showed that people also gossip to vent their emotions. Furthermore, whereas both the group protection motive and the emotion venting motive were activated when the opportunity to gossip to a victim was offered, group protection was more predictive of the tendency to gossip. In contrast, if participants had the opportunity to gossip to a non-victim, emotion venting was activated more strongly and it was more predictive of the tendency to gossip than group protection.

These findings provide further evidence for the bright side of gossip, indicating that people gossip because they are motivated to protect their group members from harm (e.g., Gluckman, 1963; Beersma and Van Kleef, 2011). This corresponds to the argument that gossip is a prosocial act (Feinberg et al., 2012a, 2012b) and resonates with gossip’s acclaimed functional role in maintaining group cooperation, also in real-world team contexts (e.g., Dunbar, 2004; Kniffin and Wilson, 2010; Feinberg et al., 2014; Wu et al., 2016a, 2016b). However, our findings also demonstrate that gossip is not always driven by prosocial motives to protect the group but might also serve people’s emotional balance by venting emotions (e.g., Waddington and Fletcher, 2005; Georganta et al., 2014).

Although we found that the group protection motive was most strongly activated when gossip recipients were potential victims of norm violators, we found that this motive was also activated even if the recipients did not run the risk of being harmed by the gossip target. This could indicate that regardless of the people involved, group protection is a strong driver of gossip when norm violations are experienced. It appears that people aim to warn group members of norm violators by harming the violators’ reputation for future interactions, and the recipients of gossip may spread this further. This corresponds with indirect reciprocity, where in networks, free-riders’ reputations spread leading to their exclusion (Nowak and Sigmund, 2005).

The finding that emotion venting also played a role when gossiping to recipients who might become a victim of the norm violator could indicate emotion venting is an additional and strong driver of gossip regardless of whether this is beneficial to the recipient or not (e.g., Grosser et al., 2012; Georganta et al., 2014). Feinberg et al. (2012b) already reported that gossip about norm violations reduces the negative affect caused by such norm violations, and our findings show this may be even more the case when gossiping to a non-victim of a norm violator. Corroborating this idea, our results showed that gossip motives explained more variance in the tendency to gossip in the non-victim condition when people had no prosocial reasons to gossip but primarily did so in order to vent their emotions. Emotional venting can be argued to be a more selfish motive than good protection, as it is aimed at feeling better while harming the reputation and feelings of the target. Such more selfishly motivated (negative) gossip has been argued to lead to detrimental consequences for groups because it might reduce trust and group cohesion (see for example Paine, 1967; McAndrew et al., 2007; Melwani, 2012). Yet it remains unclear if and how individual gossip motives relate to group outcomes. We will return to this issue in the Section “General Discussion.”

## Study 2

Whereas Study 1 highlighted the “bright” side of gossip, we argue that gossip might also have a dark side. Research has shown that most people can remember gossip they heard about themselves (Martinescu, 2017). But previous experimental studies on gossip ignored how people react to gossip, particularly in those cases where it is unjustified. Wu et al. (2018) recently stressed the importance of the gossip target’s perspective to highlight the negative aspects of gossip, demonstrating that awareness of negative gossip about oneself related to lower organizational citizenship behavior through decreased organization-based self-esteem. Furthermore, Xie et al. (2018) showed that being the target of negative gossip related to less proactive behavior and increased emotional exhaustion. Furthermore, being the target of negative gossip related to increased intentions to repair social relationships through self-directed blame (Martinescu, 2017).

Furthermore, people do not always truthfully gossip about third parties; false gossip has been argued to occur to a non-trivial extent (Smith, 2014; Fonseca and Peters, 2018) and could have detrimental consequences such as increased cynicism (Kuo et al., 2015) and aggressive responses from the target (Kuttler et al., 2002; Giardini, 2012). Although the current scientific image of gossip is that it is an effective and low-cost form of punishment of non-cooperation in groups (Feinberg et al., 2012b), false gossip could work much like a false punishment. Research on punishment of cooperative individuals (antisocial punishment) shows that false punishment reduces a target’s cooperation after the punishment (Herrmann et al., 2008).

Finally, we argue that the focus of previous gossip research has narrowly focused on short-term cooperation in social dilemma games as gossip’s outcome. When gossip is argued to be good for groups, it is important to define what constitutes “good outcomes.” In real-world organizational settings, broader outcomes than short-term cooperation in social dilemmas are important. Gossip research has ignored more distal and less visible indicators of cooperation, such as long-term cooperative intentions. Findings by Beersma and Van Kleef (2011) demonstrated that the threat that group members might gossip about non-cooperation increased cooperation with the group if cooperative acts were identifiable by group members, but not if participants’ choices for whether to cooperate or not remained private. This might indicate that gossip causes compliance to norms of cooperation, but not intrinsic motivation for cooperation. If gossip would stimulate compliance to cooperative norms in the short run (because people fear that they might become the target of gossip), but at the same time cause the potential breakdown of long-term cooperation, it may not be so functional for organizational groups after all.

We argue that people who realize that there is gossip about them could experience the gossip as a form of punishment (Feinberg et al., 2012b) and therefore as an external incentive to cooperate. External incentives to cooperate have been shown to deteriorate trust in group members, to lower intrinsic cooperative intentions, and to harm future cooperative interactions once the external threat is removed (Mulder et al., 2006; Chen et al., 2009; Nelissen and Mulder, 2013). If this is the case, gossip could be especially damaging for organizational teams in the long run.

In order to examine the effects of gossip on both short-term and long-term cooperative intentions, in Study 2, we present a scenario study that (1) examines the effects of gossip on targets as well as on observers of gossip, (2) examines effects of gossip veracity, and (3) takes into account a broader time interval in addition to short-term cooperation. We operationalize short-term cooperation as work effort, the extent to which group members are willing to invest in their group tasks immediately (De Cremer and Van Knippenberg, 2002; Kacmar et al., 2007). We operationalize long-term cooperation as cooperative intentions toward gossiping group members; the extent to which group members are willing to collaborate with gossipers in future tasks (Rigby et al., 1997; Jap, 2001).

In line with earlier findings on cooperation as a result of gossip threat, we first predicted a main effect of gossip target versus gossip observer on short term cooperation, such that gossip targets would intend to exert more work effort than gossip observers (H2a). Second, we predicted that the main effect of target versus observer is qualified by gossip veracity, such that gossip only leads to increased work efforts of targets when the gossip is true rather than false (H2b).

Furthermore, following from earlier findings on extrinsic incentives for long term cooperation, we predicted a main effect of target versus observer, such that gossip targets would display lower long-term cooperative intentions toward gossipers than gossip observers (H3a). We do not make predictions about the effects of gossip veracity on long term cooperative intentions but test these effects exploratively.

### Methods

#### Participants

A total of 104^4^ participants who were students at a large public university (79.6%), at a university of applied sciences (18.4%), or who followed vocational education (2.0%) in the Netherlands were recruited via email and social media to participate in a 2 (target of gossip versus observer of gossip) by 2 (gossip is true versus false) between-subjects design. Table 4 reports demographic variables and their distribution across the conditions. There were no differences in demographics between the conditions (*p*s > 0.068, Cramer’s *V* < 0.26).

**TABLE 4:** Descriptive statistics of age, gender, and education across conditions.

	**True gossip**				**False gossip**				**Combined**			
	***N***	***M*_age_ (*SD*_age_)**	**% Female**	**% University**	***N***	***M*_age_ (*SD*_age_)**	**% Female**	**% University**	***N***	***M*_age_ (*SD*_age_)**	**% Female**	**% University**
Target	25	22.48	80.0%	84.0%	26	23.88	73.1%	61.5%	51	23.20	76.5%	72.5%
		(2.12)				(3.77)				(3.13)		
Observer	27	22.70	74.1%	85.2%	26	23.16	56.0%	84.6%	53	22.92	65.4%	84.9%
		(1.46)				(2.56)				(2.01)		
Combined	52	22.60	76.9%	84.6%	52	23.53	64.7%	74.5%				
		(1.80)				(3.23)						

#### Procedure and Scenarios

Following the invitation to participate in a study concerning people’s participation in teamwork (we again avoided the word “gossip” because of social desirability concerns) via email, Facebook or Twitter, participants received a link to a Dutch online questionnaire if they had indicated they wanted to participate. Participants read a short introduction explaining that the study concerned people’s participation in teamwork, and they started the questionnaire with the SVO measure. After this, participants were randomly assigned to read one of four possible combinations of the target/observer and veracity scenarios. Each participant read a scenario in which she had to imagine working in a group of 4 students: Lisa (common female first name in Dutch), Daan, Thijs (common male first names in Dutch), and themselves. The project involved a presentation of the work they had completed thus far. The scenario described that the participant was waiting in front of the lecture hall when Lisa and Daan walked into the hallway and were unaware of the participant’s presence. Lisa and Daan were discussing the project and gossiped about one of the other group members slacking off. In the target condition, the participant heard Lisa telling Daan that she was annoyed about the participant not investing the required effort into the task and Daan agreed that the participant did not do his/her best. In the observer condition, the participant heard Lisa telling Daan that she was annoyed about Thijs (the other group member) not investing the required effort into the task; Daan agreed that Thijs did not do his best. In the false condition, participants were to imagine thinking back of the energy and time they/Thijs had invested in the project and to realize they/Thijs had actually invested a lot of time and had truly done their best. In the true condition, participants were told to imagine thinking back to the energy and time they/Thijs invested in the project and to realize they/Thijs had indeed invested little time in the project and did not really do their best. Participants were randomly assigned to one of four possible combinations of conditions. When participants had read the scenario, they completed the emotion questionnaire followed by the questionnaires measuring attitudes, work effort, trust in the group, commitment, and cooperative intentions.^5^ After that, participants provided their thoughts on the goal of the study and demographics. Most participants did not answer this question, those that gave answers were in line with teamwork and/or cooperation, fitting with the recruitment information. Finally, participants were thanked for their participation and were debriefed about the actual goal of the study regarding gossip. The study protocol was approved by the ‘Research Ethics Review Committee (RERC)’ of the Social Sciences faculty at the Vrije Universiteit Amsterdam.

#### Measures

##### Work effort

Participants were asked to indicate the work effort they would intend to invest in the project after overhearing the gossip using 5 items on 5-point Likert scale ranging from 1 (*completely disagree*) to 5 (*completely agree*) from the Work-Effort Questionnaire (e.g., “I would try to work harder during the remainder of the project.”; Kacmar et al., 2007). The mean of the items formed a composite score that had adequate internal consistency, α = 0.74. Descriptive statistics per condition can be seen in Table 5. Overall scores ranged from 1 to 5 (*M* = 3.42, *SD* = 0.85).

**TABLE 5:** Means (standard deviations) for work effort and control questions.

	**True**			**False**			**Combined**		
	**Work effort**	**Check true**	**Check target**	**Work effort**	**Check true**	**Check target**	**Work effort**	**Check true**	**Check target**
Target	$4.11{}^{\text{ab}}$	3.44⁢b	$4.00{}^{\text{a}}$	$3.27{}^{\text{b}}$	$2.19{}^{\text{ab}}$	$4.13{}^{\text{a}}$	$3.68{}^{\text{a}}$	2.81	$4.07{}^{\text{a}}$
	(0.75)	(0.73)	(0.34)	(0.65)	(0.63)	(0.80)	(0.81)	(0.93)	(0.61)
Observer	$3.21{}^{\text{a}}$	3.48⁢b	$1.95{}^{\text{a}}$	3.15	$2.70{}^{\text{ab}}$	$1.81{}^{\text{a}}$	$3.18{}^{\text{a}}$	3.11	$1.89{}^{\text{a}}$
	(0.87)	(0.58)	(0.75)	(0.77)	(0.64)	(0.81)	(0.81)	(0.72)	(0.77)
Combined	$3.64{}^{\text{b}}$	$3.46{}^{\text{b}}$	2.92	$3.21{}^{\text{b}}$	$2.44{}^{\text{b}}$	2.97			
	(0.92)	(0.65)	(1.19)	(0.71)	(0.68)	(1.42)			

*Control questions are the composite of items measuring whether participants correctly interpreted the scenario with regard to who was the target of gossip and whether the gossip was true or false. Superscripts indicate within condition comparisons (all *p*s < 0.018). ^a^Significant difference between observer and target. ^b^Significant difference between true and false.*

##### Cooperative intentions

Participants were asked to indicate their cooperative intentions toward the gossiping group members (Daan and Lisa) and to the non-gossiping group member (Thijs) using 15 items with a 5-point Likert scale ranging from 1 (*completely disagree*) to 5 (*completely agree*). The items were adapted from the Cooperativeness scale (Rigby et al., 1997) to fit the current scenario. An exploratory principal components analysis with direct oblimin rotation (see Supplementary Table 1) was performed on the selected and adapted items from the Cooperativeness scale. Four factors with an eigenvalue larger than 1 were identified (minimum eigenvalue = 1.27; total variance explained = 76.82%). The factors correspond to cooperative intentions toward the group members involved in the gossip (e.g., “I would like to work with Lisa/Daan.”; α = 0.92; *M* = 2.63, *SD* = 0.67), cooperative intentions toward Thijs (e.g., I would like to get to know Thijs better; α = 0.87; *M* = 3.03, *SD* = 0.69), intentions with regards to sharing ideas (e.g., “I would rather not share my ideas with Lisa/Daan/Thijs.”; α = 0.87; *M* = 3.44, *SD* = 0.82), and preference to work alone (e.g., “I would find it more productive to do this project by myself.”; α = 0.85; *M* = 3.26, *SD* = 1.05). We focus on cooperative intentions toward Lisa/Daan and toward Thijs in the results section, as these are most central to our hypothesis on how gossip affects long-term cooperative intentions. Results for the other factors are described in the Supplementary Material. Descriptive statistics per condition for cooperative intentions can be found in Table 6.

**TABLE 6:** Means and standard deviations for the cooperative intentions subscales.

	**True**				**False**				**Combined**			
	**Daan/Lisa**	**Thijs**	**Share Ideas**	**Work Alone**	**Daan/Lisa**	**Thijs**	**Share Ideas**	**Work Alone**	**Daan/Lisa**	**Thijs**	**Share Ideas**	**Work Alone**
Target	$2.47{}^{\text{a}}$	3.07	$3.16{}^{\text{a}}$	3.34	$2.34{}^{\text{a}}$	2.99	3.44	3.12	$2.40{}^{\text{a}}$	3.03	3.30	3.23
	(0.64)	(0.71)	(0.73)	(1.06)	(0.70)	(0.69)	(0.97)	(1.09)	(0.66)	(0.70)	(0.86)	(1.07)
Observer	$2.94{}^{\text{a}}$	$2.79{}^{\text{b}}$	$3.62{}^{\text{a}}$	3.13	$2.76{}^{\text{a}}$	$3.28{}^{\text{b}}$	3.53	3.48	$2.85{}^{\text{a}}$	3.03	3.57	3.30
	(0.64)	(0.76)	(0.79)	(0.98)	(0.58)	(0.52)	(0.76)	(1.08)	(0.61)	(0.70)	(0.77)	(1.03)
Combined	2.72	2.92	3.39	3.23	2.55	3.13	3.48	3.30				
	(0.67)	(0.74)	(0.79)	(1.01)	(0.67)	(0.62)	(0.86)	(1.09)				

*Superscripts indicate within condition comparisons (all *p*s < 0.018). ^a^Significant difference between observer and target. ^b^Significant difference between true and false.*

##### Control questions

Participants answered several control questions regarding the scenarios, which were all rated on Likert scales ranging from 1 (*completely disagree*) to 5 (*completely agree*). There were three questions regarding gossip veracity (e.g., “In the scenario, the gossip by Lisa and Daan was true.”) and three questions about who the target was (e.g., “In the scenario, there was gossip about me/Thijs.”). Both sets of items were combined to form a composite score with adequate reliability (α_true_ = 0.78, α_victim_ = 0.93). The gossip veracity scores ranged from 1 to 4.33 (*M* = 2.97, *SD* = 0.83) and the scores on the target versus observer scale ranged from 1 to 5 (*M* = 2.95, *SD* = 1.30). Descriptive statistics per condition can be seen in Table 5.

##### Other measures

As in Study 1, participants completed the Triple Dominance Measure (Van Lange et al., 1997) to measure SVO; analyses including SVO are presented in the Supplementary Material. Furthermore, participants also filled out the Dutch PANAS inventory (Watson et al., 1988; Peeters et al., 1996) to measure their positive and negative affect after reading the gossip scenario, and a number of measures of their reactions to the scenario. For brevity, exploratory analyses on positive and negative affect and reactions to the scenario are presented in the Supplementary Material. Participants also indicated what they thought the goal of the study was. Lastly, they were asked about their age, gender, education level, education program, whether they had a job, and for how many hours a week they worked in that job.

#### Statistical Analyses

To test hypotheses 2a and 2b, a 2 × 2 full factorial ANOVA was performed to test the effects of target manipulation (gossip target versus observer) and gossip veracity (true versus false) on work effort.^6^

To test hypotheses 3a and 3b, a 2 × 2 full factorial ANOVA was performed to test the effects of target condition (gossip target versus observer) and veracity condition (true versus false) on cooperative intentions toward Lisa and Daan and toward Thijs.

### Results

The correlations between demographics, target condition, veracity condition, work effort, and manipulation checks can be found in Table 7. Most of the cooperative intentions subscales correlated positively amongst one another, indicating individuals who have cooperative intentions in one dimension also have cooperative intentions in another dimension. Cooperative intentions toward Daan and Lisa were positively associated with cooperative intentions toward Thijs (*r* = 0.30) and the intentions to share ideas (*r* = 0.35), while they were negatively associated with preference to work alone (*r* = −0.35). Preference to work alone was negatively associated with intention to share ideas (*r* = −0.37). Further, work effort was not associated with any of the cooperative intentions subscales.

**TABLE 7:** Intercorrelations between demographics, dependent and independent variables (Study 2).

	**1**	**2**	**3**	**4**	**5**	**6**	**7**	**8**	**9**	**10**
**Cooperative intentions:**										
(1) With Daan and Lisa										
(2) With Thijs	$0.30{}^{**}$									
(3) Work alone	$-0.35{}^{*,**}$	–0.19								
(4) Share ideas	$0.35{}^{*,**}$	0.07	$-0.37{}^{*,**}$							
(5) Work effort	–0.06	–0.03	0.03	< 0.01						
(6) Veracity condition	0.13	–0.15	–0.03	–0.05	$0.26{}^{**}$					
(7) Target -condition	$-0.33{}^{**}$	< 0.01	–0.04	–0.17	$0.30{}^{**}$	–0.02				
(8) Gender	–0.19	–0.04	0.13	–0.08	0.19	0.13	0.12			
(9) Age	0.01	–0.12	–0.09	–0.03	–0.02	–0.18	0.05	$-0.21{}^{*}$		
(10) Education	–0.03	0.01	–0.06	–0.03	< 0.01	0.13	0.17	0.01	$-0.28{}^{**}$	

Veracity condition is coded 1 = true, 0 = false, target condition is coded 1 = target, 0 = observer, SVO is coded 1 = prosocial, 0 = proself, gender is coded 1 = male, 0 = female, education is coded 1 = university of applied sciences, 0 = university. ^*^p < 0.05, ^∗∗^*p* < 0.01, ^∗∗∗^*p* < 0.001.

#### Manipulation Checks

Results of the control questions indicated that individuals in the gossip target condition more strongly agreed that that they themselves, rather than someone else, were the gossip targets in the scenario, *F*(1,70) = 176.83, *p* < 0.001, ${\text{η}}_{\text{p}}^{\text{2}}$ = 0.72, indicating a large effect size^7^ (see Table 5). The veracity condition did not differ on the composite of questions about the target, *F*(1,70) < 0.01, *p* = 0.987, ${\text{η}}_{\text{p}}^{\text{2}}$ < 0. 01, nor did the manipulations interact, *F*(1,70) = 0.65, *p* = 0.423, ${\text{η}}_{\text{p}}^{\text{2}}$ = 0.01. Conversely, individuals in the true condition scored higher on the veracity questions composite compared to individuals in the false condition, which means that they indicated to a higher extent that gossip was true, *F*(1,70) = 46.28, *p* < 0.001, ${\text{η}}_{\text{p}}^{\text{2}}$ = 0.40, indicating a large effect size (see Table 6). Targets and observers of gossip did not differ on the composite of questions about veracity, *F*(1,70) = 3.46, *p* = 0.067, ${\text{η}}_{\text{p}}^{\text{2}}$ = 0.05, nor did the manipulations interact, *F*(1,70) = 2.56, *p* = 0.114, ${\text{η}}_{\text{p}}^{\text{2}}$ = 0.04. These results indicate that both manipulations were successful.

#### Hypothesis Testing

##### Work effort

Supporting Hypothesis 2a, there was a medium to large main effect of the target manipulation; gossip targets indicated that they intended to make significantly more work efforts than observers, see Table 5, *F*(1,100) = 11.78, *p* = 0.001, ${\text{η}}_{\text{p}}^{\text{2}}$ = 0.11. This indicates people intend to put more effort into group work after being the target of gossip compared to observing gossip that is directed at another group member.

There was also a medium to large main effect of veracity condition where false gossip led to lower work effort intentions than true gossip, see Table 6, *F*(1,100) = 9.11, *p* = 0.003, ${\text{η}}_{\text{p}}^{\text{2}}$ = 0.08. This indicates that people intend to put more effort into group work when the gossip was true compared to false.

**FIGURE 2:**
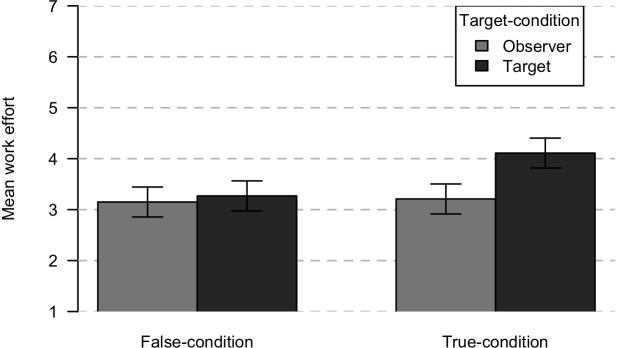
Means for intended work effort after true or false gossip depending on whether the participant is an observer or the target. Error bars represent 95% confidence intervals.

Supporting Hypothesis 2b, these main effects were qualified by a significant interaction effect, *F*(1,100) = 6.81, *p* = 0.010, ${\text{η}}_{\text{p}}^{\text{2}}$ = 0.06, which indicates a medium effect. This interaction is depicted in Figure 2. To further examine this interaction, simple effects analysis was used. Results showed that in the true condition, there was a significant difference between observers of gossip in work effort for the group (*M* = 3.21, *SD* = 0.87) compared to targets of gossip (*M* = 4.11, *SD* = 0.75), *F*(1,100) = 18.23, *p* < .001, ${\text{η}}_{\text{p}}^{\text{2}}$ = 0.15, indicating the difference was large. In contrast, in the false condition, there was no significant difference between observers (*M* = 3.15, *SD* = 0.77) and targets of gossip (*M* = 3.27, *SD* = 0.65), *F*(1,100) = 0.34, *p* = 0.562, ${\text{η}}_{\text{p}}^{\text{2}}$ < 0.01. This result indicates that the influence of being the gossip target versus an observer depends on whether the gossip is true or false. Individuals are more likely to put more effort into the group work only when they are the target of true gossip.

##### Cooperative intentions

*Toward the gossiping group members (Daan and Lisa).* Supporting Hypothesis 3a, there was a main effect of target condition indicating that gossip targets had lower cooperative intentions toward Lisa and Thijs (*M* = 2.40, *SD* = 0.66) than observers (*M* = 2.85, *SD* = 0.61), *F*(1,100) = 12.59, *p* = 0.001, ${\text{η}}_{\text{p}}^{\text{2}}$ = 0.11. The effect was medium to large. This indicates that people intend to cooperate less with the gossiping group members after they have been the targets of gossip themselves compared to when they observed gossip targeting another group member.

There was neither a significant main effect of veracity condition, *F*(1,100) = 1.65, *p* = 0.202, ${\text{η}}_{\text{p}}^{\text{2}}$ = 0.02, nor an interaction effect between the factors, *F*(1,100) = 0.31, *p* = 0.860, ${\text{η}}_{\text{p}}^{\text{2}}$ < 0.01. These results indicate that being the gossip target or an observer predicts an individual’s intention to cooperate with the gossiping group members, whereas her cooperative intentions are unaffected by whether the gossip is true or false.

**FIGURE 3:**
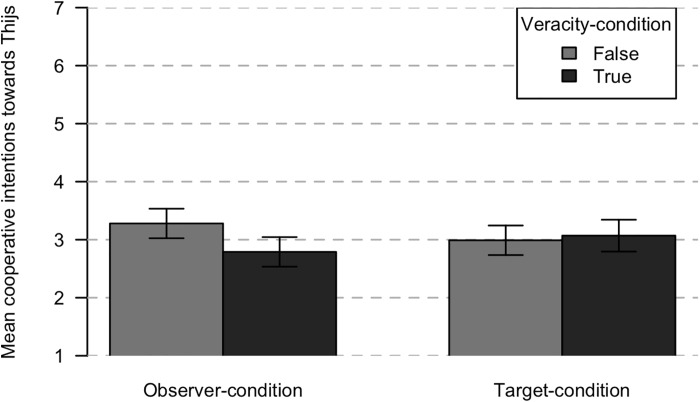
Means for cooperative intentions toward Thijs after true or false gossip depending on whether the participant is an observer or the target. Error bars represent 95% confidence intervals.

*Cooperative intentions toward non-gossiping group member (Thijs).* A 2 by 2 full factorial ANOVA was performed to test the effect target condition (gossip target versus observer) and true condition (true versus false) on cooperative intentions toward Thijs showed no significant main effect of gossip veracity, *F*(1,100) = 2.41, *p* = 0.124, ${\text{η}}_{\text{p}}^{\text{2}}$ = 0.02. There was also no significant main effect of target condition, *F*(1,100) = 0.01, *p* = 0.945, ${\text{η}}_{\text{p}}^{\text{2}}$ < 0.01. However, results did show a small to medium significant interaction effect between the factors, *F*(1,100) = 4.63, *p* = 0.034, ${\text{η}}_{\text{p}}^{\text{2}}$ = 0.04. This interaction is depicted in Figure 3. To further examine this interaction, simple effects analysis was used. Results showed that in the observer condition, there was a medium difference in the cooperative intentions toward Thijs when gossip was true (*M* = 2.79, *SD* = 0.76) compared to when it was not true (*M* = 3.28, *SD* = 0.52), *F*(1,100) = 15.54, *p* < 0.001, ${\text{η}}_{\text{p}}^{\text{2}}$ = 0.14. In contrast, in the target condition, there was no significant difference between true (*M* = 3.07, *SD* = 0.71) and false gossip (*M* = 2.99, *SD* = 0.69), *F*(1,100) = 0.08, *p* = 0.676, ${\text{η}}_{\text{p}}^{\text{2}}$ < 0.01. Participants intended to reduce their cooperation with the gossip target if they had an indication that the gossip about the alleged uncooperativeness of the gossip target was true. This could reflect indirect reciprocity, as the likelihood that people will work with Thijs increases after false rather than true gossip.

Further, neither veracity of gossip nor target condition or their interaction affected participants’ preference to work alone and willingness to share ideas. For complete results see Supplementary Material.

### Discussion

Our second study aimed to test the effects of being the target or an observer of gossip – both in terms of immediate work effort and general willingness to cooperate in the future. Furthermore, we analyzed how true gossip, as opposed to false gossip, affects targets differently. In line with our hypotheses, targets of gossip compared to observers intended to put more effort into work for their groups when they became aware of true, rather than false, gossip about themselves. At the same time, however, they reported lower intentions than observers of gossip to cooperate with their gossiping group members in the long run. Notably, gossip targets only reported on increased work effort when the gossip was true but not when it was false. The effect of being a target or observer of gossip on long-term cooperative intentions was not influenced by gossip veracity; those who were confronted with being the target of gossip were less inclined to cooperate with their group in the long run, no matter whether the gossip was true or false.

Our findings regarding immediate work effort in reaction to gossip support the perspective of a bright side of gossip by showing that in the short term gossip can increase intentions to work harder toward group goals. This is in line with findings showing that gossip targets want to repair social relationships and increase their cooperation to become part of the group again (Beersma and Van Kleef, 2011; Feinberg et al., 2014; Martinescu et al., 2014). However, this bright side seems to be dependent on specific aspects of gossip, as it only emerges when group members find out they are the target of true gossip about their norm-violating behavior. When gossip was false or concerned another group member, participants’ short-term cooperation did not increase. This could indicate that many instances of real-life gossip do not affect group outcomes positively and could, in fact, have negative consequences.

Even if the gossip is true, our results suggest that it has negative consequences in the long run. Those affected by gossip reported lower intention to cooperate with the gossiping group members in the future regardless of whether the gossip was true or false. Apparently, people perceive gossip as punishment (Feinberg et al., 2012b). Such punishment presents an external incentive to cooperate but seems to deteriorate intrinsic motivation to engage in cooperation (Fehr and Falk, 2002; Mulder et al., 2006; Chen et al., 2009; Nelissen and Mulder, 2013). This means that groups engaging in negative gossip could gain short-term benefits, but they could bear the long-term costs when team cooperation breaks down.

Furthermore, the evidence for diminished intention to cooperate after realizing that one has become the gossip target oneself– regardless of gossip veracity – indicates that this effect is likely to occur frequently in the real world. This dark side of gossip could be especially detrimental for team-based organizations, where gossip is omnipresent (e.g., Grosser et al., 2012) and where long-term cooperation with the same team members is essential for the organization to thrive (Che and Yoo, 2001; Ferres et al., 2004).

Taken together, these findings corroborate the importance of the target’s perspective and of taking into account broader dependent variables than short-term cooperation for understanding the effects of gossip (Wu et al., 2018). This resonates with findings by Martinescu (2017), Xie et al. (2018), and Martinescu et al. (under review). Together, these recent findings highlight that gossip research needs to analyze more long-term and broader outcomes than immediate reactions in order to illuminate the dark side of gossip that has been overlooked in much of the earlier literature on gossip.

## General Discussion

The current paper contributes to developing a complete understanding of both positive and negative aspects of gossip in groups. We make the following four contributions to the field of gossip research. First, we demonstrated that gossip can be driven by different motives in different situations: Group protection motivated gossip more when gossip was directed to a potential victim of a norm violator, whereas emotion venting motivated gossip more when gossip was directed to a non-victim recipient. Second, we demonstrated that the perspective of the target can provide insight into both positive and negative aspects of gossip. Third, we demonstrated that considering false gossip can nuance current insights into when gossip has positive consequences. Finally, we demonstrated that considering a broader range of outcomes including long-term cooperative intentions can illuminate the negative consequences of gossip for groups. The latter three contributions follow from the findings that being the target of true, but not false, gossip increases short-term cooperation with one’s group, yet being the target of gossip (whether the gossip is right or wrong) reduces long-term cooperative intentions toward the group. Taken together, the results indicate that gossip has both a dark side and a bright side for groups, and both situational factors (i.e., who is the gossip recipient and is the gossip true or false) and agent perspectives (being the target or the observer of gossip) determine which side prevails.

The bright side of gossip as a behavior that benefits groups seems to prevail when there is an opportunity to gossip to a potential victim of a norm violator. Warning group members about norm violators through gossip can be seen as a prosocial act that allows cooperators to consult together and ensures that norm violators must cooperate to be part of the group (Apicella et al., 2012; Feinberg et al., 2014; Giardini et al., 2014). These benefits only seem to occur when the gossip is really about norm violators because accurate information allows for selecting cooperative interaction partners (Laidre et al., 2013), whereas false gossip seems to weaken the power of gossip to increase cooperation (Seki and Nakamaru, 2016; Fonseca and Peters, 2018). Finally, when there is true gossip about norm violations, the bright side of gossip prevails from the perspective of the sender and recipient, as gossip carries benefits for both parties. The recipient directly benefits from being protected from exploitation; the sender could benefit from venting their emotions and reducing negative affect (Waddington and Fletcher, 2005; Feinberg et al., 2012b; Nils and Rimé, 2012). Also, gossipers might benefit from a potentially higher prosocial status (Willer, 2009) because others could perceive a gossip sender as a prosocial person when they benefit others by warning them about free-riders (Baumeister et al., 2004; Grosser et al., 2010; Feinberg et al., 2012a, 2012b). At the same time, being known as a gossiper can deter free-riders from exploiting the gossiper as this could have detrimental consequences for the free-rider (Piazza and Bering, 2008; Beersma and Van Kleef, 2011; Feinberg et al., 2012b).

From the perspective of the gossip target, the dark side of gossip seems to be dominant; worse, over time, it may hurt entire groups as well. Being the target of either true or false negative gossip reduces group members’ intrinsic cooperative intentions toward their group. Gossip can be interpreted as punishment (Feinberg et al., 2012a) and therefore presents an extrinsic incentive to cooperate and avoid more punishment, but it lowers trust in the punishers and willingness to cooperate with these group members (e.g., Fehr and Falk, 2002; Mulder et al., 2006). Further, being the target of (true or false) negative gossip induced negative affect and negative attitudes toward the group (see Supplementary Material). Being the target of negative gossip can be painful through signaling lack of inclusion, which can lead to aggression and non-cooperation (Twenge et al., 2007; DeWall et al., 2012; Martinescu et al., under review). Additionally, being the target of false gossip could lead to costly retaliation that negatively affects the overall group (Kuttler et al., 2002; Giardini, 2012).

On top of this, it is possible that gossip aimed at non-victims of others’ norm-violating behavior could also be part of a dark side of gossip. We found that such gossip is mostly motivated by emotion venting, which could be argued to be a selfish motive to reduce one’s own negative affect (Cialdini et al., 1987; Fehr and Gächter, 2002). In the case where gossip recipients correctly perceive that gossip is driven by such more selfish motives, they could judge this unfavorably and this, in turn, could have negative consequences for group cohesion and functioning (Fiske et al., 2007; Brandt et al., 2011; Beersma and Van Kleef, 2012).

Our findings imply that gossip research would benefit from integrating these insights on both the bright and dark side of gossip as they are intertwined in a single gossip instance. Thus, gossip is not only good for groups but can also be a bad thing and vice versa. As the situation and perspective determine whether the bright or dark side of gossip will become more salient, it is imperative for future research to consider the situation in which gossip takes place and to decide on which actor (gossip sender, recipient, or target) to focus.

Our findings also have implications for real-world organizations dealing with gossip. While it is not possible to prevent gossip (Ben-Ze’ev and Goodman, 1994; Ribeiro and Blakely, 1995), gossip could be managed to lead to positive outcomes. Therefore, we would advise managers to see the presence of gossip as a signal that there could be a situation that requires intervention. By gathering information about the motives driving specific instances of gossip and its veracity, managers can gain a perspective on what is going on in their team and use this to decide whether to intervene to ensure positive group outcomes and diminish negative outcomes.

### Limitations and Strengths

Our studies had several limitations. Firstly, sample sizes were small, which could increase the risk of false-positive results and could have obscured smaller effects, such as interactive effects (Cohen, 1992; Maxwell, 2004). Therefore, the current results should be interpreted with caution and require further testing.

Second, both of our studies were scenario studies. While scenarios allow for embedding gossip in a real-world context, it is possible that behavioral intentions diverge from actual behaviors (Ajzen et al., 2004). Future research could use behavioral measures of both gossip and (long term) cooperation in laboratory, or more ideally, in (longitudinal) field settings. In such studies, it is of vital importance to take measures that protect study participants from unethical consequences, as gossip is obviously a sensitive process.

Furthermore, people could have responded in a socially desirable manner. For example, it would be considered socially desirable to protect the recipient from the norm violator when they can become a victim, while social enjoyment might be considered less desirable in this situation. While this could have influenced the results, social desirability is unavoidable when investigating motives, as self-reports are required. However, we observed a not-insignificant tendency to gossip in both conditions, alleviating this was a concern for our participants. Future research could measure socially desirable answering (e.g., Crowne and Marlowe, 1960) to investigate whether it affects gossip motives and tendencies.

Finally, the current studies exclusively relied on students that could be characterized as a WEIRD sample (White, Educated, Industrialized, Rich, and Democratic; Henrich et al., 2010). Therefore, we cannot be sure whether our results generalize to the majority of employees in modern organizations nor to the general population. However, it is important to note that the goals of the current study were not to demonstrate effects that could be directly transferred to other contexts, but rather to show that both a bright and dark side of gossip can emerge when the situation affords it and that our hypothesized effects present themselves in these specific experimental situations (Mook, 1983; Ilgen, 1985).

Despite these limitations, the current studies had several strengths. The scenarios used represented gossip and norm violations in an intuitive and real-life manner. This allowed participants to more closely report how they would respond in real life compared to more abstract measures such as economic games. Since the dynamics of gossip are difficult and ethically sensitive to analyze, the current scenarios offered the possibility to examine them as precisely as possible. Furthermore, the current studies redress gaps in the previous literature and provide a methodology that can be used and adapted in future research, such as specifying the scenarios for organizational samples. Finally, the current studies take a first step in combining the perspectives that gossip could have a bright and a dark side, as well as the perspective of all involved parties, thereby integrating literatures that have up to now developed separately.

### Future Research Directions

As our studies provide preliminary insights into the dark side of gossip in addition to the bright side, there are several avenues for future research to pursue. The dark side of gossip especially requires further research. One possible direction for future research could be to investigate whether people’s cooperative intentions change after they have discovered negative or positive gossip about themselves without explicitly knowing which group members have gossiped about them. Another direction could be to examine situations that activate other gossip motives such as social enjoyment, information gathering and validation, and negative influence. It is possible that these motives could play a larger role when gossip does not concern norm violators. For example, the social enjoyment motive could particularly be activated for gossip about a mutual acquaintance because it presents a shared interest (Foster, 2004); negative influence could be more likely to motivate gossip when there is an ongoing conflict or when the conflict between the involved parties is more severe (Jeuken et al., 2015).

While the current studies highlight the role of the situation in determining the motives and consequences of gossip, individual characteristics could also play a role in determining gossip behavior (Lewin, 1936; Lyons and Hughes, 2015). Future research could investigate the role of individual differences in personality such as agreeableness and honesty-humility, which are found to be important in both active and reactive cooperation decisions (Ashton and Lee, 2007; Hilbig et al., 2015) for both gossip senders and targets. For example, realizing that one is the target of negative gossip or false gossip might be less detrimental when one is high on agreeableness, which relates to being more forgiving following defection (Hilbig et al., 2016).

Additionally, the current study emphasizes the role of negative gossip in groups. While this type of gossip is important for detecting free-riders through passing on negative information about their reputation (e.g., Feinberg et al., 2012b), people also share positive information, for example, about previous cooperation (Foster, 2004; Sommerfeld et al., 2007). It has been argued that reputation systems built on positive gossip such as rewards or positive reviews are more common in real-life and more efficient in promoting cooperation (Wu et al., 2016b). Perceiving positive gossip about oneself was related to increased intentions to improve oneself, increased intentions to affiliate, and increased happiness (Martinescu et al., 2014; Martinescu, 2017). This indicates positive gossip could function similarly to negative gossip without activating the dark side of gossip. Future research could investigate positive gossip and its effects on cooperation in groups and compare this to negative gossip.

Furthermore, the current research leaves open the puzzle of whether specific motives for gossiping and group outcomes in terms of cooperation are related. Therefore, future research should aim to integrate the investigation of gossip motives at the individual level with group level gossip behavior and outcomes to illuminate whether some motives (e.g., group protection) lead to specific consequences (e.g., benefits) or whether gossip from any one motive can both harm and benefit groups (Beersma et al., 2019).

## Conclusion

In conclusion, we aimed to investigate both the bright and dark side of gossip and support both the current consensus in the gossip literature that gossip is a good thing for groups as well as more scattered arguments that gossip can be a bad thing for groups. Integrating these bright and dark sides of gossip by addressing limitations in previous research, we showed that situational and perspective-related factors determine which side of gossip prevails. Therefore, it becomes imperative for research and practice to consider the situation that gossip occurs in and whether one is focusing on the sender, recipient, or target when interpreting and studying gossip. It seems that gossip remains a paradoxical behavior that has both positive and negative aspects. Integrative insights on gossip can aid in fully understanding the phenomenon of gossip, which is an essential and pervasive element of all human groups and can be key in solving challenges of cooperation such as working in teams in the workplace.

## Ethics Statement

This study was carried out in accordance with the recommendations of Code of Ethics for Research in the Social and Behavioral Sciences Involving Human Participants, Research Ethics Review Committee (RERC) of the Faculty of Social Sciences at the VU University Amsterdam with written informed consent from all subjects. All subjects gave written informed consent in accordance with the Declaration of Helsinki. The protocol was approved by the Research Ethics Review Committee (RERC).

## Author Contributions

TDC analyzed the data and wrote the first draft of the manuscript. BB designed the studies and coordinated data collection. BB, MD, MB, and TDC commented and worked on sections of the manuscript.

## Conflict of Interest Statement

The authors declare that the research was conducted in the absence of any commercial or financial relationships that could be construed as a potential conflict of interest.

## Footnotes

1 Compared to the original version (Beersma and Van Kleef, 2011), in this version of the questionnaire, small changes in the wording of several items were made (e.g., “For me, a reason to instigate this conversation was to protect the person I was talking with against the person we were talking about was” changed to “For me, a reason to instigate this conversation was to protect the person I was talking with against the behavior of the person we were talking about.”).

2 Participants were recruited from a pool of students at a campus of University students and University of Applied Sciences students. Therefore, we coded for these two educational levels.

3 All analyses yielded similar results when controlling for age, education, and gender.

4 Demographics were missing for one participant. The manipulation check was added later in the study and is missing for 29 participants.

5 Analyses using attitudes, commitment, and trust in the group are reported in the Supplementary Material.

6 All analyses provided similar results when controlling for age, education, and gender.

7 Degrees of freedom differ from other analyses due to 29 missing values from participants who did not complete the manipulation check that was added later during data collection.
