# Supplementary material for: The Bright and Dark Side of Gossip for Cooperation in Groups
Source: Front Psychol. 2019 Jun 20;10:1374. doi: 10.3389/fpsyg.2019.01374 (PMC6596322; doi:10.3389/fpsyg.2019.01374)
Supplement: Supplementary file 1 [file Data_Sheet_1.docx]

Supplementary Material

# Study 1

## Method

Social value orientation (SVO) was measured using the Triple Dominance Measure (Van Lange, Otten, DeBruin, & Joireman, 1997). This measure consists of nine items in which participants make a prosocial, individualistic, or competitive choice in a decomposed social dilemma game. For example, participants decide between option A (you get 480, the other gets 80; competitive choice), option B (you get 540, the other gets 280; individualistic choice), and option C (you get 480, the other gets 480; prosocial choice). Participants were classified into a category if they made six consistent choices. Most participants were categorized as prosocial (61; 56.5%), 32 (29.6%) were categorized as proselfs, 3 (2.8%) were categorized as competitors, and 12 participants could not be categorized. As is customary in the SVO literature, the competitive and individualistic categories were combined to form the proself category (35; 32.4%). The participants who were not categorized were excluded from the analyses involving SVO.

## Results

The correlations between SVO, the motives to gossip, the victim-condition and demographics can be found in Supplementary Table 2.

We used a moderated mediation regression analysis to test whether motives to gossip (information gathering and validation, social enjoyment, negative influence, emotion ventilation, and group protection; mediator variables) mediated the relationship between victim-condition (dummy-coded 1 = victim, 0 = non-victim; predictor variable) and tendency to gossip (outcome variable).In addition, we wanted to test whether the relationship between gossip receiver condition and the motives to gossip was moderated by social value orientation (SVO; dummy-coded 1 = prosocial, 0 = proself). We performed these tests using the software package PROCESS (Hayes, 2013; Model 7) and tested the significance of the indirect effect with bias-corrected bootstrapping procedures using 10.000 samples^^[[1]](#footnote-1)^^. All variables, except for victim-condition and SVO, were standardized prior to being entered into the model.

The victim-condition had a medium to large positive effect on the tendency to gossip (total effect: *B* = 0.71, *se* = 0.19, *t*(94) = 3.65, *p* < .001, Cohen’s *d* = 0.75), indicating that when the target of gossip is a potential victim, people were more likely to gossip than when the target is an observer. The effects of the gossip receiver condition, SVO, and their interaction in predicting each motive can be seen in Supplementary Table 3. When controlling for SVO and the interaction between a motive and SVO, the victim-condition had a large positive effect on the group protection motive, indicating those in the victim-condition were more likely to support the group protection motive for gossip. The victim-condition had a large negative effect on the negative influence motive, indicating those in the victim condition were less likely to support the negative influence motive for gossip. SVO had a large negative effect on the negative influence motive, indicating prosocials were less likely to support the negative influence motive for gossip.

Solely the effects of the victim-condition and SVO on the negative influence motive were qualified by a significant interaction effect with a large effect size. We further investigated, this effect using simple slopes analyses, which showed that for proselfs there was a large negative effect of the gossip receiver condition, *B* = -0.61, *se* = 0.30, *t*(34) = -2.04, *p* = .044, Cohen’s *d* = -3.05. In contrast, for prosocials, there was a large positive effect of the gossip receiver condition, *B* = 0.45, *se* = 0.22, *t*(60) = 2.02, *p* = .046, Cohen’s *d* = 2.94. This indicates that proselfs were less likely to support the negative influence motive when they could gossip to a potential victim (*M* = 2.14, *SE* = 0.24) versus when they could not (*M* = 2.81, *SE* = 0.22), whereas prosocials were more likely to support the negative influence motive when they can gossip to a potential victim (*M* = 2.51, *SE* = 0.17) versus when they could not (*M* = 2.01, *SE* = 0.17), see Figure S1.

When controlling for the victim-condition and the other motives, the emotion ventilation motive (*B* = 0.40, *se* = 0.09, *t*(89) = 4.52, *p* < .001, partial *r*^2^ = .43) and the group protection motive (*B* = 0.39, *se* = 0.11, *t*(89) = 3.47, *p* = .001, partial *r*^2^ = .35) had a positive effect on the tendency to gossip, both with large effect sizes. When controlling for the victim-condition and the other motives, the social enjoyment motive (*B* = 0.10, *se* = 0.09, *t*(89) = 1.12, *p* = .266, partial *r*^2^ = .12), information gathering and validation motive (*B* = -0.03, *se* = 0.10, *t*(89) = -.25, *p* = .801, partial *r*^2^ = -.03), and the negative influence motive (*B* = -0.07, *se* = 0.11, *t*(89) = -0.66, *p* = .511, partial *r*^2^ = -.07) had no significant effect on the tendency to gossip.

After controlling for all motives, the relationship between the victim-condition and the tendency to gossip was weaker and no longer significant (direct effect: *B* = 0.39, *se* = 0.23, *t*(89) = 1.70, *p* = .092, partial *r*^2^ = .18) This indicates that the gossip motives fully mediate the relationship between being able to gossip to a potential victim of the target (or not) on the tendency to gossip. The bootstrapped indirect effects of each motive per condition can be found in Supplementary Table 4. The indirect effect of the negative influence motive was non-significant in neither condition. This indicates that while SVO influences the effect of being able to gossip to a potential victim (or not) on the negative influence motive, this does not, in turn, predict the tendency to gossip.

While there was an overall mediation effect of emotion ventilation, there was no statistically significant indirect effect of emotion ventilation for proselfs or prosocials separately. Lastly, there was a statistically significant indirect effect of the gossip receiver condition on the tendency to gossip through the group protection motive for both proself and prosocials. While the effect appears to be larger for prosocials, the difference was not statistically significant. This indicates that the mediation by group protection is not significantly influenced by SVO.

# Study 2

## Method

### Social Value Orientation

Social value orientation (SVO) was measured as in Study 1 using the Triple Dominance Measure (Van Lange, et al., 1997). Most participants were categorized as prosocial (51; 49.0%), 32 (30.8%) were categorized as proselfs, 4 (4.8%) were categorized as competitors, and 16 participants could not be categorized. As is customary in the SVO literature, the competitive and individualistic categories were combined to form the proself category (37; 35.6%). The participants that were not categorized were excluded from the analyses involving SVO.

### PANAS

Participants were asked about their positive and negative affect using the Dutch PANAS-scale (Peeters, Ponds, & Vermeeren, 1996), for which participants indicated on a five-point Likert scale to what extent 20 emotions were applicable to them (1 = very little, 2 = a little, 3 somewhat, 4 = a lot, 5 = very much). The scale consists of a positive affect dimension (e.g., “enthusiastic”, α = .77) with scores ranging from 1.10 to 4.40 (*M* = 2.69, *SD* = 0.60) and a negative affect dimension (e.g., “upset”, α = .91), with scores ranging from 1 to 4.70 (*M* = 2.56, *SD* = 0.92).

### Negative attitude towards the group

Participants were asked to indicate their negative attitudes towards the group using 10 self-constructed items fitting the current scenario using a 5-point Likert scale ranging from 1 (*completely disagree*) to 5 (*completely agree*). The scale consisted of items such as “I would be angry when I hear Lisa and Daan talk like this”^[[2]](#footnote-2)^. The scale showed good internal consistency (α = .86) with scores ranging from 1.40 to 5 (*M* = 3.21, *SD* = 0.73).

### Commitment towards the group

Participants were asked to indicate their commitment towards the group using the team-commitment component of the three-component model of commitment (De Gilder, van den Heuvel & Ellemers, 1997). The scale consists of 7 items using a 5-point Likert scale ranging from 1 (*completely disagree*) to 5 (*completely agree*). An example is “This group would be very important for me”. The scale showed low internal consistency (α = .68) with scores ranging from 1 to 4.43 (*M* = 3.27, *SD* = 0.53).

### Trust towards the Group

Participants were asked to indicate their trust towards the gossiping group members (Daan and Lisa) and towards the non-gossiping group member (Thijs) using 15 items with a 5-point Likert scale ranging from 1 (*completely disagree*) to 5 (*completely agree*). The items were adapted from the Trust Questionnaire (Jarvenpaa & Leidner, 1999) to fit the current scenario. An exploratory principal components analysis with direct oblimin rotation (see Supplementary Table 5) was performed on the selected and adapted items from the Trust Questionnaire. Three factors with an eigenvalue larger than 1 were identified (minimum eigenvalue = 1.62; total variance explained = 69.40%). The factors correspond to trust towards the group members involved in the gossip (e.g., “I think Lisa/Daan is trustworthy.”; α = .88; *M* = 2.61, *SD* = 0.67), trust towards Thijs (e.g., I think Thijs is trustworthy; α = .71; *M* = 3.05, *SD* = 0.67), trusting others with the assignment (e.g., “I would not mind giving Lisa/Daan/Thijs complete responsibility for this project.”; α = .89; *M* = 2.55, *SD* = 0.77,).

## Results

The correlations between SVO, cooperative intentions, negative intentions towards the group, commitment towards the group, trust towards the group, target-condition, veracity-condition, and demographics can be found in Supplementary Table 6. There was a significant positive association between age and positive affect, which indicates that older participants felt more positive affect than younger participants. There was a significant negative association between gender and negative affect; men felt less negative affect (*M* = 2.23, *SD* = 0.90) than women (*M* = 2.70, *SD* = .91). Further, negative affect was positively associated with cooperative intentions towards Daan and Lisa, but it was negatively associated with the willingness to share ideas. This indicates that negative affect is differently related to different aspects of cooperative intentions.

### Cooperative intentions

#### Preference to work alone

A 2 by 2 full factorial ANOVA was performed to test the effect of the target-condition (gossip target versus observer) and the true-condition (true versus false) on the preference to work alone, which showed no significant main effect of the true-condition (*F*(1,100) = 0.09, *p* = .760, partial *η*^2^ < .01^[[3]](#footnote-3)^),no significant main effect of the target-condition (*F*(1,100) = 0.14, *p* = .708, partial *η*^2^ < .01), and no significant interaction effect between the factors (*F*(1,100) = 1.95, *p* = .166, partial *η*^2^ = .02). This indicates that the manipulations had no influence on participants’ preference to work alone.

#### Willingness to share ideas

A 2 by 2 full factorial ANOVA was performed to test the effect of the target-condition (gossip target versus observer) and the true-condition (true versus false) on the willingness to share ideas, which showed no significant main effect of the true-condition (*F*(1,100) = 0.33, *p* = .568, partial *η*^2^ < .01),no significant main effect of the target-condition (*F*(1,100) = 2.90, *p* = .092, partial *η*^2^ = .03), and no significant interaction effect between the factors (*F*(1,100) = 1.31, *p* = .255, partial *η*^2^ =.01). This indicates that the manipulations had no influence on the willingness to share ideas with the other group members.

### Positive Affect

A 2 by 2 full factorial ANOVA was performed to test the effect of the target-condition (gossip target versus observer) and the true-condition (true versus false) on positive affect. There was a medium to large main effect of the true-condition. True gossip (*M* = 2.55, *SE* = 0.08) led to significantly lower positive affect than false gossip (*M* = 2.83, *SE* = 0.08), *F*(1, 100) = 5.64 , *p* = .020, partial *η*^2^ = .05. This indicates that participants who heard true gossip felt less positively than those who heard false gossip. There was no effect of the target-condition (*F*(1, 100) = 1.97, *p* = .164, partial *η*^2^ = .02) and no significant interaction effect between the factors (*F*(1,100) = 1.61, *p* = .208, partial *η*^2^ = .02).

### Negative affect

A 2 by 2 full factorial ANOVA was performed to test the effect of the target-condition (gossip target versus observer) and the true-condition (true versus false) on negative affect. A large main effect of the target-condition revealed that gossip targets (*M* = 3.12, *SE* = 0.10) had a higher negative affect than gossip observers (*M* = 2.03, *SE* = 0.10), *F*(1,100) = 54.99, *p* < .001, partial *η*^2^ = .36. This indicates that participants who were the target of gossip felt more negative affect than those who observed gossip about another person. There was no significant main effect of the true-condition (*F*(1,100) = 0.03, *p* = .867, partial *η*^2^ < .01) and no significant interaction effect between the factors (*F*(1,100) = 0.85, *p* = .358, partial *η*^2^ = .01).

### Negative attitudes towards the group

A 2 by 2 full factorial ANOVA was performed to test the effect of the target-condition (gossip target versus observer) and the true-condition (true versus false) on negative attitudes towards the group. A medium to large main effect of the true-condition showed that true gossip (*M* = 3.05, *SE* = 0.08) led to significantly lower negative attitudes towards the group than false gossip (*M* = 3.38, *SE* = 0.08), *F*(1, 100) = 7.61, *p* = .007, partial *η*^2^ = .07. This indicates that participants who heard false gossip reported a more negative attitude towards the group than those that heard true gossip. A large main effect of the target-condition revealed that gossip targets (*M* = 3.59, *SE* = 0.09) had higher negative attitudes towards the group than observers (*M* = 2.84, *SE* = 0.08), *F*(1,100) = 40.17, *p* < .001, partial *η*^2^ = .29. This indicates that participants who were the target of gossip reported more negative attitudes towards the group than those who observed gossip about another person. There was no significant interaction effect between the factors, *F*(1,100) = 0.22, *p* = .641, partial *η*^2^ < .01.

### Commitment to the group

A 2 by 2 full factorial ANOVA was performed to test the effect of the target-condition (gossip target versus observer) and true-condition (true versus false) on commitment to the group. There was no significant main effect of the true-condition (*F*(1, 100) = 3.01, *p* = .086, partial *η*^2^ = .03), no significant main effect of the target-condition (*F*(1,100) = 0.64, *p* = .424, partial *η*^2^ = .01), and no significant interaction effect between the factors (*F*(1,100) = 0.56, *p* = .641, partial *η*^2^ = .01).

### Trust towards the group

#### Trust in the gossiping group members (Daan and Lisa)

A 2 by 2 full factorial ANOVA was performed to test the effect of the target-condition (gossip target versus observer) and the true-condition (true versus false) on trust towards the gossiping group members (Daan and Lisa). A medium main effect of the true-condition revealed that true gossip (*M* = 2.76, *SE* = 0.09) led to significantly higher trust towards the gossiping group members than false gossip (*M* = 2.45, *SE* = 0.08), *F*(1, 100) = 6.28, *p* = .014, partial *η*^2^ = .06. This indicates that participants who heard false gossip trusted the gossiping group members less than those who heard true gossip. There was a medium main effect of the target-condition. Gossip targets (*M* = 2.45, *SE* = 0.09) had significantly lower trust towards the gossiping group members than observers (*M* = 2.76, *SE* = 0.08), *F*(1,100) = 5.95, *p* = .016, partial *η*^2^ = .06. This indicates that participants who were the target of gossip reported a lower trust negative attitude towards the gossiping group members than those who observed gossip about another person. There was no significant interaction effect between the factors, *F*(1,100) = 1.00, *p* = .320, partial *η*^2^ = .01.

#### Trusting others with the assignment

A 2 by 2 full factorial ANOVA was performed to test the effect of the target-condition (gossip target versus observer) and the true-condition (true versus false) on trusting others with the assignment. There was no significant main effect of the true-condition (*F*(1, 100) = 2.68, *p* = .105, partial *η*^2^ = .03), T no significant main effect of the target-condition (*F*(1,100) = 0.19, *p* = .667, partial *η*^2^ < .01), and no significant interaction effect between the factors, *F*(1,100) = 0.58, *p* = .449, partial *η*^2^ = .01.

#### Trust in the non-gossiping group member (Thijs)

A 2 by 2 full factorial ANOVA was performed to test the effect of the target-condition (gossip target versus observer) and the true-condition (true versus false) on trust towards the non-gossiping group member (Thijs). There was no significant main effect of the true-condition (*F*(1, 100) = 0.93, *p* = .337, partial *η*^2^ = .01), no significant main effect of the target-condition (*F*(1,100) = 1.20, p = .277, partial *η*^2^ = .01), and no significant interaction effect between the factors, *F*(1,100) = 0.01, *p* = .945, partial *η*^2^ < .01.

### Social Value Orientation

We ran all models tested above and in the main text including SVO as a factor and testing for a three-way interaction between SVO and the conditions as factors.

SVO did not influence work effort (*F*(1,80) = 0.64, *p* = .428, partial *η*^2^ = .01) nor did it interact with the veracity-condition (*F*(1,80) = 0.73, *p* = .394, partial *η*^2^ = .01) or the target-condition (*F*(1,80) = 0.07, *p* = .795, partial *η*^2^ < .01), nor was there a three-way interaction between these factors (*F*(1,80) = 0.08, *p* = .772, partial *η*^2^ < .01).

SVO did not influence cooperative intentions (*F*(1,80) = 0.47, *p* = .494, partial *η*^2^ = .01) nor did it interact with the veracity-condition (*F*(1,80) = 1.66, *p* = .202, partial *η*^2^ = .02) or the target-condition (*F*(1,80) = 0.03, *p* = .40, partial *η*^2^ < .01), nor was there a three-way interaction between these factors (*F*(1,80) = 0.30, *p* = .585, partial *η*^2^ < .01).

SVO did not influence cooperative intentions towards Thijs (*F*(1,80) = 0.83, *p* = .366, partial *η*^2^ =.01) nor did it interact with the veracity-condition (*F*(1,80) = 0.43, *p* = .512, partial *η*^2^ = .01) or the target-condition (*F*(1,80) = 0.01, p = .931, partial *η*^2^ < .01), nor was there a three-way interaction between these factors (*F*(1,80) = 3.81, *p* = .055, partial *η*^2^ = .05).

SVO did not influence cooperative intentions towards Lisa and Daan (*F*(1,80) = 0.17, *p* = .678, partial *η*^2^ =.01) nor did it interact with the veracity-condition (*F*(1,80) = 0.56, *p* = .457, partial *η*^2^ = .01) or the target-condition (*F*(1,80) = 0.31, *p* = .580, partial *η*^2^ < .01), nor was there a three-way interaction between these factors (*F*(1,80) = 0.22, *p* = .641, partial *η*^2^ < .01).

SVO did not influence the preference to work alone (*F*(1,80) = 0.04, *p* = .836 partial *η*^2^ < .01) nor did it interact with the veracity-condition (*F*(1,80) = 0.29, *p* = .592, partial *η*^2^ < .01) or the target-condition (*F*(1,80) = 0.19, *p* = .669, partial *η*^2^ < .01), nor was there a three-way interaction between these factors (*F*(1,80) = .02, *p* = .878, partial *η*^2^ < .01).

There was a medium significant effect of SVO on the willingness to share ideas (*F*(1, 80) = 5.36, *p* = .023 partial *η*^2^ = .06). This indicates proselfs (*M* = 3.17, *SE* = .14) were less likely to share ideas than prosocials (*M* = 3.59, *SE* = .11). This effect was not qualified by a two-way interaction with the veracity-condition (*F*(1, 80) = 3.55, *p* = .063, partial *η*^2^ = .04) or the target-condition (*F*(1, 80) = 0.91, *p* = .342, partial *η*^2^ = .01), nor was there a three-way interaction between these factors (*F*(1, 80) < .01, *p* = .961, partial *η*^2^ < .01).

SVO did not influence positive affect (*F*(1,80) = 0.31, *p* = .580, partial *η*^2^ < .01) nor did it interact with the veracity-condition (*F*(1,80) = 0.07, *p* = .788, partial *η*^2^ < .01) or the target-condition (*F*(1,80) = 1.49, *p* = .255, partial *η*^2^ = .02), nor was there a three-way interaction between these factors (*F*(1,80) = 0.38, *p* = .541, partial *η*^2^ = .01).

SVO did not influence negative affect (*F*(1,80) = 0.17, *p* = .685, partial *η*^2^ < .01) nor did it interact with the veracity-condition (*F*(1,80) = 0.15, *p* = .697, partial *η*^2^ < .01) or the target-condition (*F*(1,80) = 0.13, *p* = .853, partial *η*^2^ < .01), nor was there a three-way interaction between these factors (*F*(1,80) = 0.04, *p* = .853, partial *η*^2^ = .01).

SVO did not influence negative attitudes towards the group (*F*(1,80) = 1.20, *p* = .277, partial *η*^2^ = .02) nor did it interact with the veracity-condition (*F*(1,80) = 0.18, *p* = .670, partial *η*^2^ < .01) or the target-condition (*F*(1,80) = 0.82, *p* = .367, partial *η*^2^ = .01), nor was there a three-way interaction between these factors (*F*(1,80) = 0.04, *p* = .841, partial *η*^2^ < .01).

SVO did not influence commitment to the group (*F*(1,80) = 0.32, *p* = .320, partial *η*^2^ = .01) nor did it interact with the veracity-condition (*F*(1,80) = 2.61, *p* = .110, partial *η*^2^ = .03) or the target-condition (*F*(1,80) = 0.02, *p* = .892, partial *η*^2^ < .01), nor was there a three-way interaction between these factors (*F*(1,80) < 0.01, *p* = .960, partial *η*^2^ < .01).

SVO did not influence trust towards the gossiping group members (*F*(1,80) = 0.27, *p* = .570, partial *η*^2^ < .01) nor did it interact with the veracity-condition (*F*(1,80) = 0.35, *p* = .110, partial *η*^2^ = .03) or the target-condition (*F*(1,80) = 3.70, *p* = .058, partial *η*^2^ = .04), nor was there a three-way interaction between these factors (*F*(1,80) = 0.04, *p* = .840, partial *η*^2^ < .01).

SVO did not influence trusting others with the assignment (*F*(1,80) = 0.80, *p* = .373, partial *η*^2^ = .01) nor did it interact with the veracity-condition (*F*(1,80) = 0.51, *p* = .477, partial *η*^2^ = .01) or the target-condition (*F*(1,80) = 0.80, *p* = .373, partial *η*^2^ = .01), nor was there a three-way interaction between these factors (*F*(1,80) = 0.25, *p* = .616, partial *η*^2^ < .01).

SVO did not influence trust towards the non-gossiping group member (*F*(1,80) = 0.21, *p* = .652, partial *η*^2^ < .01) nor did it interact with the veracity-condition (*F*(1,80) = 1.52, *p* = .221, partial *η*^2^ = .02) or the target-condition (*F*(1,80) = 0.87, *p* = .353, partial *η*^2^ = .01), nor was there a three-way interaction between these factors (*F*(1,80) = 0.98, *p* = .326, partial *η*^2^ = .01).

Most of the effects were comparable when the SVO and the interactions were added to the model. The main effect of the target-condition on the trust towards the gossiping group members was no longer statistically significant (*F*(1, 80) = 1.25, *p* = .267, partial *η*^2^ = .02). When looking at cooperative intentions towards Daan and Lisa, the effect of the veracity-condition became significant, *F*(1, 80) = 5.03, *p* = .028, partial *η*^2^ = .06, corresponding to a medium effect. This indicates that, when SVO is kept constant, people intended to cooperate more with Daan and Lisa when gossip was true (*M* = 2.83, *SE* = 0.10) compared false (*M* = 2.44, *SE* = 0.11). For the willingness to share ideas, the effect of the target-condition became significant, *F*(1, 80) = 7.04, *p* = .010, partial *η*^2^ = .08, corresponding to a medium to large effect. This indicates that, if SVO is kept constant, targets of gossip (*M* = 3.14, *SE* = .12) were less willing to sharing ideas than observers of gossip (*M* = 3.62, *SE* = .13).

# Supplementary Tables

## Supplementary Table 1.

Descriptives, factor loadings from direct oblimin rotation pattern matrix and eigenvalues for cooperative intentions.

| Dimension | Item |  | *M* | *SD* | Factor Loading | Eigenvalue  (% variance explained) |
| --- | --- | --- | --- | --- | --- | --- |
| Daan/Lisa | I would like to work together with Lisa |  | 2.65 | 0.76 | 0.83 | 5.99 (39.93) |
|  | I would like to get to know Lisa better |  | 2.83 | 0.83 | 0.79 |  |
|  | I would like to work with Lisa in a follow-up project |  | 2.52 | 0.89 | 0.88 |  |
|  | I would like to work with Daan |  | 2.65 | 0.75 | 0.77 |  |
|  | I would like to get to know Daan better |  | 2.79 | 0.81 | 0.78 |  |
|  | I would like to work with Daan in a follow-up project |  | 2,56 | 0.89 | 0.90 |  |
|  | It would be rewarding for me to work in this group |  | 2.42 | 0.80 | 0.64 |  |
| Thijs | I would like to work together with Thijs |  | 2.97 | 0.75 | 0.92 | 2.32 (15.45) |
|  | I would like to get to know Thijs better |  | 3.21 | 0.77 | 0.83 |  |
|  | I would like to work with Thijs in a follow-up project |  | 2.90 | 0.82 | 0.87 |  |
| Share ideas | I would rather not share my ideas with Lisa |  | 2.60 | 0.98 | 0.89 | 1.94 (12.93) |
|  | I would rather not share my ideas with Daan |  | 2.56 | 0.91 | 0.85 |  |
|  | I would rather not share my ideas with Thijs |  | 2.53 | 0.91 | 0.83 |  |
| Work Alone | I would rather do this project by myself |  | 3.29 | 1.14 | 0.84 | 1.27 (8.94) |
|  | I would find it more productive to do this project by myself |  | 3.24 | 1.11 | 0.93 |  |

*Note.* All factor with eigenvalue > 1 were extracted.

## Supplementary Table 2.

Correlations of SVO with motives to gossip, victim-condition, and demographics.

| Variable | 1 |
| --- | --- |
| 1 SVO |  |
| Tendency to gossip | .06 |
| Motives  Social enjoyment | .13 |
| Information gathering and validation | -.05 |
| Negative influence | -.12 |
| Emotion ventilation | -.03 |
| Group protection | .16 |
| Target-condition | .05 |
| Gender | -.06 |
| Age | -.15 |
| Education | -.07 |

## Supplementary Table 3.

Effects of gossip receiver condition, social value orientation, and their interaction on all motives to gossip.

| Outcome | Predictor | *B* | *SE* | *t* | *p* | Partial *r*^2^ |
| --- | --- | --- | --- | --- | --- | --- |
| Social enjoyment | Condition | -0.59 | 0.22 | 1.80 | .076 | -.18 |
|  | SVO | -0.05 | 0.29 | -0.19 | .849 | -.02 |
|  | Interaction | 0.69 | 0.42 | 1.68 | .096 | -.17 |
| Information gathering and validation | Condition | 0.28 | 0.32 | 0.88 | .380 | .09 |
|  | SVO | -0.44 | 0.28 | -1.61 | .112 | -.17 |
|  | Interaction | 0.65 | 0.40 | 1.64 | .106 | .17 |
| **Negative influence** | **Condition** | **-0.61** | **0.30** | **-2.04** | **.044** | **-.21** |
|  | **SVO** | **-0.72** | **0.26** | **-2.83** | **.006** | **-.28** |
|  | **Interaction** | **1.06** | **0.37** | **2.85** | **.005** | **.29** |
| Emotion ventilation | Condition | -0.35 | 0.35 | -1.01 | .315 | -.11 |
|  | SVO | -0.02 | 0.30 | -0.07 | .947 | -.01 |
|  | Interaction | -0.07 | 0.44 | -0.15 | .882 | -.02 |
| **Group protection** | **Condition** | **1.07** | **0.26** | **4.07** | **<.001** | **.39** |
|  | SVO | 0.10 | 0.23 | 0.45 | .653 | .05 |
|  | Interaction | 0.33 | 0.33 | 1.00 | .320 | .10 |

## Supplementary Table 4.

Indirect effects of gossip receiver condition on the tendency to gossip via each motive to gossip as moderated by SVO.

| Indirect effect | SVO | *B* | *SE* | 95% CI Lower bound | 95% CI Upper bound |
| --- | --- | --- | --- | --- | --- |
| Social enjoyment | Proself | -0.06 | 0.05 | -0.19 | 0.02 |
|  | Prosocial | 0.01 | 0.03 | -0.06 | 0.09 |
|  | Comparison | 0.07 | 0.07 | -0.03 | 0.23 |
| Information gathering and validation | Proself | -0.01 | 0.04 | -0.13 | 0.06 |
|  | Prosocial | -0.02 | 0.10 | -0.21 | 0.17 |
|  | Comparison | -0.02 | 0.07 | -0.16 | 0.15 |
| Negative influence | Proself | 0.04 | 0.07 | -0.09 | 0.20 |
|  | Prosocial | -0.03 | 0.05 | -0.13 | 0.08 |
|  | Comparison | -0.08 | 0.11 | -0.29 | 0.16 |
| Emotion ventilation | Proself | -0.14 | 0.14 | -0.43 | 0.11 |
|  | Prosocial | -0.17 | 0.11 | -0.37 | 0.06 |
|  | Comparison | -0.03 | 0.17 | -0.36 | 0.34 |
| **Group protection** | **Proself** | **0.42** | **0.18** | **0.11** | **0.81** |
|  | **Prosocial** | **0.55** | **0.25** | **0.14** | **1.11** |
|  | Comparison | 0.13 | 0.15 | -0.09 | 0.49 |

*Note.* Gossip receiver condition is dummy coded (0 = non-victim, 1 = victim).

## Supplementary Table 5

Descriptives, factor loadings from direct oblimin rotation pattern matrix and eigenvalues for Trust towards the group

*Note.* All factor with eigenvalue > 1 were extracted.

| Dimension | Item |  | *M* | *SD* | Factor Loading | Eigenvalue  (% variance explained) |
| --- | --- | --- | --- | --- | --- | --- |
| Daan/Lisa | I think Lisa is nice |  | 2.63 | 0.78 | 0.86 | 5.77 (38.45) |
|  | I think Daan is nice |  | 2.62 | 0.79 | 0.86 |  |
|  | In this group, there is no trust between us (R) |  | 2.41 | 0.91 | 0.75 |  |
|  | I think Daan is trustworthy |  | 2.71 | 0.82 | 0.75 |  |
|  | I think Lisa is trustworthy |  | 2.74 | 0.85 | 0.71 |  |
|  | There is no team spirit in our group (R) |  | 2.54 | 0.90 | 0.68 |  |
|  | I would trust each other’s work |  | 2.88 | 0.85 | 0.45 |  |
| Thijs | I think Thijs is trustworthy |  | 3.00 | 0.79 | 0.89 | 1.62 (10.78) |
|  | I think Thijs is nice |  | 3.11 | 0.74 | 0.78 |  |
| Others Responsible | I would not mind having Daan hand in the final version, even if I could then not check it anymore |  | 2.51 | 1.01 | 0.87 | 3.03 (20.17) |
|  | I would not mind having Lisa hand in the final version, even if I could then not check it anymore |  | 2.61 | 1.06 | 0.87 |  |
|  | I would not mind giving Daan complete responsibility for this project |  | 2.42 | 1.05 | 0.84 |  |
|  | I would not mind giving Lisa complete responsibility for this project |  | 2.50 | 1.08 | 0.81 |  |
|  | I would not mind having Thijs hand in the final version, even if I could then not check it anymore |  | 2.46 | 0.98 | 0.67 |  |
|  | I would not mind giving Thijs complete responsibility for this project |  | 2.43 | 1.03 | 0.66 |  |

## Supplementary Table 6.

Intercorrelations between SVO, positive affect, negative affect, cooperative intentions, work effort, negative attitudes towards the group, commitment to the group, trust towards the group, target-condition, veracity-condition, and demographics.

|  | 1 | 2 | 3 | 4 | 5 | 6 | 7 | 8 | 9 | 10 | 11 | 12 |
| --- | --- | --- | --- | --- | --- | --- | --- | --- | --- | --- | --- | --- |
| 1 SVO |  |  |  |  |  |  |  |  |  |  |  |  |
| 2 Positive Affect | -.01 |  |  |  |  |  |  |  |  |  |  |  |
| 3 Negative Affect | -.02 | -.02 |  |  |  |  |  |  |  |  |  |  |
| Cooperative intentions  4 With Daan and Lisa | .02 | .12 | -.46*** |  |  |  |  |  |  |  |  |  |
| 5 With Thijs | .08 | -.06 | -.02 | .30** |  |  |  |  |  |  |  |  |
| 6 Work alone | .03 | .03 | .05 | -.35*** | -.19 |  |  |  |  |  |  |  |
| 7 Share ideas | -.20 | -.02 | -.30** | .35*** | .07 | -.37*** |  |  |  |  |  |  |
| 8 Work Effort | -.06 | -.03 | .44*** | -.06 | -.03 | .03 | <.01 |  |  |  |  |  |
| 9 Veracity-condition | .16 | -.27** | -.03 | .13 | -.15 | -.03 | -.05 | .26** |  |  |  |  |
| 10 Target-condition | -.10 | -.10 | .59*** | -.33** | <.01 | -.03 | -.17 | .30** | -.02 |  |  |  |
| 11 Gender | -.07 | -.19 | .22* | .06 | -.04 | .13 | -.08 | .19 | .13 | .12 |  |  |
| 12 Age | -.17 | .31** | -.09 | .01 | -.12 | -.09 | -.03 | -.02 | -.18 | .05 | -.21* |  |
| 13 Education | .22* | -.17 | -.18 | .11 | .01 | .07 | .07 | .01 | .18 | -.22* | -.09 | -.36*** |

*Note.* * *p* < .05, ** *p* < .01, *** *p* < .001

# Supplementary Figures


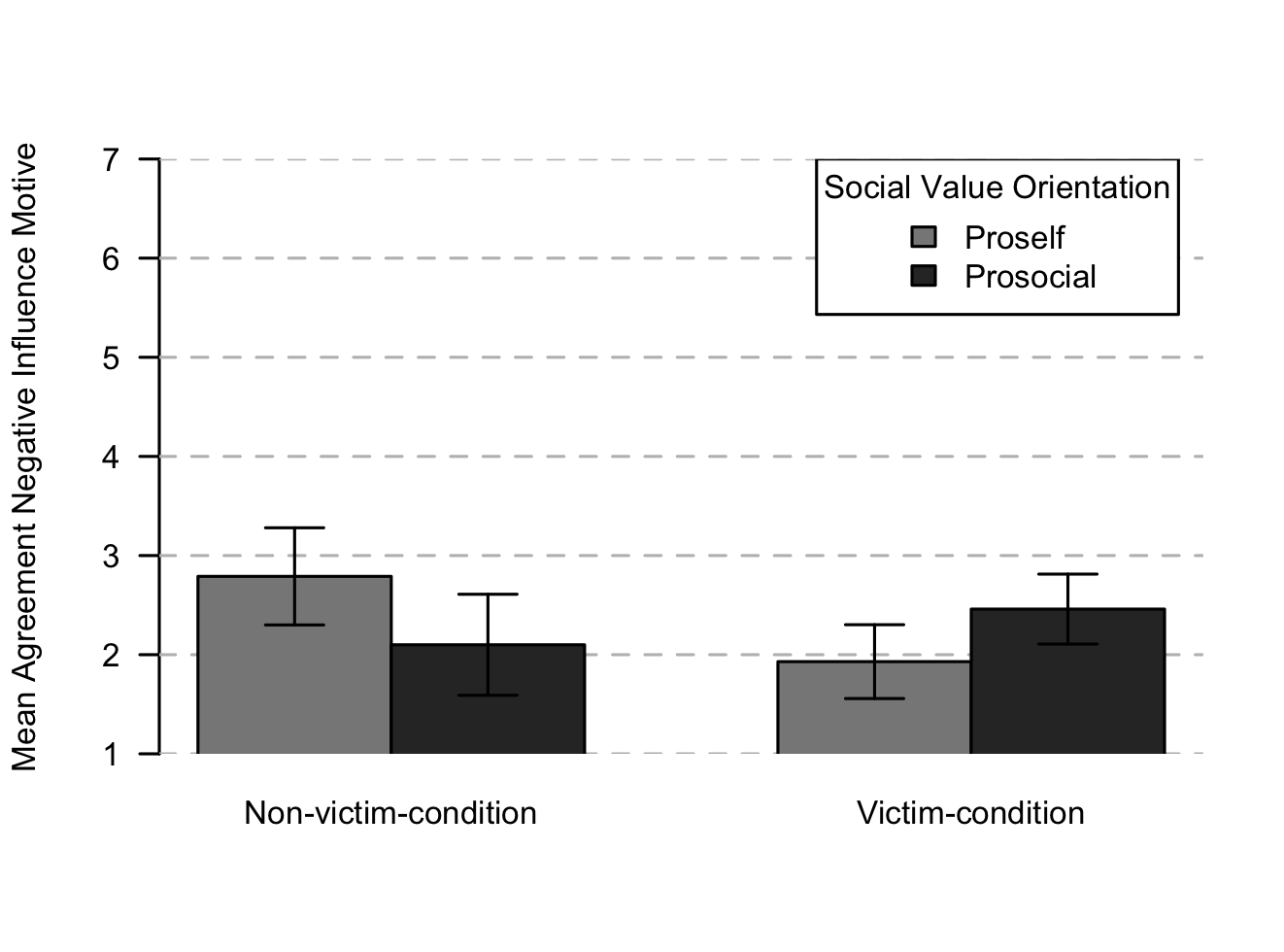


**Supplementary Figure 1.** Mean agreement with the negative influence motive grouped per gossip victim condition and per SVO. Error bars represent 95% confidence intervals.

1. All analyses yielded similar results when controlling for age and gender. However, when controlling for age, gender, and education the direct of gossip target condition was weaker but remained significant, *B*(*se*) = 0.50 (0.23), *t*(86) = 2.19, *p* = .031. This indicates partial mediation as opposed to full mediation. [↑](#footnote-ref-1)
2. Complete scale: “I would feel excluded in this group.”; I would find it painful to hear Lisa and Daan talk like this.”; “It would make me happy to be able to work in this group.” (R); I would feel at home in this group. (R)”; I would get angry when I hear Lisa and Daan talk like this.”; I would find it frustrating to work in this group.”; I would be indignant when I hear Lisa and Daan talk like this.’; I would feel unsafe in this group.”; I would be nervous when I have to talk to Lisa and Daan.”; I would find it scary to talk to Lisa and Daan in the future.”. [↑](#footnote-ref-2)
3. Most analyses yielded similar results when controlling for age, education, and gender. The effect of the true-condition on commitment became significant, *F*(1, 96) = 4.28, *p* = .041, partial *η*^2^ =.04. This shows that participants who heard true (*M* = 3.37, *SE* = 0.07) compared to false (*M* = 3.15, *SD* = 0.08) gossip indicated more commitment to the group. [↑](#footnote-ref-3)
